# Engineered AcrIIA5 for optogenetic control of CRISPR‐Cas9‐based genome editing

**DOI:** 10.1002/mlf2.70016

**Published:** 2025-12-12

**Authors:** Qi Chen, Jia Yao, Yingfan Lu, Ruikang Qiu, Zixin Deng, Yuhui Sun

**Affiliations:** ^1^ Key Laboratory of Combinatorial Biosynthesis and Drug Discovery (Wuhan University) Ministry of Education, and Wuhan University School of Pharmaceutical Sciences Wuhan China; ^2^ School of Pharmacy Huazhong University of Science and Technology Wuhan China

**Keywords:** anti‐CRISPR, CRISPR‐Cas9, optogenetics, spatiotemporal control

## Abstract

The CRISPR‐Cas9 system has been proven to be a powerful tool for gene editing in living cells and shows great potential in genetic disease treatment. Anti‐CRISPR (Acr)‐based optogenetic tools could spatiotemporally regulate the activity of CRISPR‐Cas9, thereby improving the precision and safety of gene editing. However, these tools could only regulate a certain Cas9 protein because of the high specificity of Acr used, limiting their further application. In this study, we developed a new optogenetic tool named CASANOVA‐A5 (CRISPR‐Cas9 activity switching via a novel optogenetic variant of AcrIIA5) by inserting the blue light sensor AsLOV2 into AcrIIA5 with a broad inhibition spectrum. We proved that the CASANOVA‐A5 could regulate the gene editing activity of SpCas9, SaCas9, NmeCas9, and St1Cas9 in a blue light‐dependent manner. Additionally, we engineered AcrIIA5‐LOV9 by integrating the blue light‐dependent degron module LOV9, showing obvious optical regulation for SpCas9. Together, our work demonstrates two feasible methods to engineer the Acrs to potent optogenetic tools and suggests systematic strategies for further optimization.

## Impact statement

The bacterial‐derived CRISPR‐Cas9 system has been widely applied in life sciences due to its high programmability. To reduce the toxic side effects of this system, various spatiotemporal induction methods have been developed. Among these, the light‐sensitive induction system based on anti‐CRISPR (Acr) proteins showed high sensitivity. However, these systems were based on Acrs with narrow inhibition spectra, limiting their ability to regulate the growing number of other Cas9 proteins. This study developed a series of light‐sensitive AcrIIA5 variants that can broadly inhibit various Cas9 systems that were widely used. Furthermore, this study provides a detailed examination of the effectiveness of amino acid mutations in the light sensor, offering valuable insights for the development and optimization of other light‐sensitive Acrs.

## INTRODUCTION

The CRISPR‐Cas system is an essential adaptive immune defense system for bacteria and archaea^1^. Through engineering, this system could facilitate efficient and precise manipulation of genomic DNA^2^, ^3^, ^4^. Notably, the Class 2 type II CRISPR system, which utilizes a single effector Cas9 protein with guide RNA (gRNA) to target specific gene loci, has been applied in the fields of genetic disease treatment^5^, ^6^, ^7^, epigenetic modifications^8^, ^9^, ^10^, and transcriptional regulation^11^, ^12^, ^13^.

The SpCas9 (type II‐A, 1368 aa, NGG PAM, from *Streptococcus pyogenes*) remains the most extensively utilized Cas9 protein. Nevertheless, the substantial size of SpCas9 has impeded its further application in clinical therapy, given that adeno‐associated virus (AAV), the predominant gene therapy vector, has a strict packaging limit of 4.5 kb. With the continued discovery of new CRISPR systems, an increasing number of type II Cas9 orthologs with smaller size recognizing different PAMs have been identified and modified into gene editing tools. Among them, SaCas9 (type II‐A, 1053 aa, NNGRRT PAM, from *Staphylococcus aureus*)^14^, NmeCas9 (type II‐C, 1081 aa, NNNNGATT PAM, from *Neisseria meningitidis*)^15^, and St1Cas9 (type II‐A, 1121 aa, NNAGAA PAM, from *S. thermophilus*)^16^ have been packaged into a single AAV vector and have shown high editing efficiency^14^, ^17^, ^18^. Undoubtedly, these novel Cas9 orthologs are poised to play crucial roles in future gene therapy.

However, the CRISPR‐Cas9 system has been encountering a serious challenge—occasionally, the sgRNA with Cas9 proteins may erroneously target non‐designated locus, resulting in severe off‐target effects^19^, ^20^. To improve the safety of the CRISPR‐Cas9 system, researchers have developed several inducible strategies aiming to limit the lifespan of Cas9 proteins by reducing the exposure time of the genome to Cas9^21^, ^22^, ^23^.

In general, chemically inducible CRISPR‐Cas9 systems require the addition of specific chemicals such as rapamycin^21^, doxycycline^22^, and 4‐hydroxytamoxifen^23^. However, these inducers have shown potential cytotoxicity and cannot be rapidly removed once used, posing unpredictable risks^24^, ^25^, ^26^. By contrast, light‐inducible CRISPR‐Cas9 systems are noninvasive and easy to switch off, providing precise temporal and spatial control^27^, ^28^, ^29^, ^30^, ^31^, ^32^, ^33^, ^34^. These systems can be broadly classified into two categories based on whether Cas9 itself is modified. The first category involves splitting Cas9 and fusing it to a light‐induced dimerization system, such as the blue light‐controlled paCas9^27^ system and the far‐red light‐controlled far‐red light (FRL)–activated split‐Cas9 (FAST)^30^ system. The second category involves modifying exogenous proteins that can influence Cas9 activity, thereby indirectly regulating Cas9 function through light control. Among them, the blue light‐inducible CRISPR‐Cas9 systems based on anti‐CRISPR (Acr), a bacteriophage‐derived antagonist of CRISPR‐Cas systems, offered convenient and efficient blue light induction with high sensitivity, proving to be promising optogenetic tools^31^, ^32^, ^33^, ^34^.

Specifically, the CASANOVA (CRISPR‐Cas9 activity switching via a novel optogenetic variant of AcrIIA4)^31^, LICASINO^32^, and OPERA4^33^ systems can regulate the activity of SpCas9 in a blue light‐dependent manner. Additionally, CASANOVA‐C3^34^ was capable of regulating the activity of NmeCas9 in a similar blue light‐dependent fashion. Nevertheless, the AcrIIA4 and AcrIIC3 used above can only inhibit a specific Cas9 protein, meaning that the light‐inducible systems developed based on them are generally ineffective for other Cas9 proteins. Therefore, when discovering new Cas9 orthologs, researchers must redesign corresponding Acr proteins, which is undoubtedly time‐consuming and labor‐intensive. Recently, AcrIIA5 was identified and proven to be a broad‐spectrum inhibitor capable of potently inhibiting diverse Cas9 orthologs of type II‐A, type II‐B, and type II‐C^35^, ^36^, ^37^. Hence, it is speculated that the AcrIIA5‐based light‐inducible CRISPR‐Cas9 system could be applied in regulating numerous Cas9 orthologs of type II.

In this study, we engineered the CASANOVA‐A5 (CRISPR‐Cas9 activity switching via a novel optogenetic variant of AcrIIA5), created by embedding the AsLOV2 into AcrIIA5. CASANOVA‐A5 showed strong inhibition on SpCas9, SaCas9, NmeCas9, and St1Cas9 in the dark state while restoring the activity of Cas9 proteins upon blue light irradiation. Additionally, we engineered a novel blue light‐sensitive and degron‐based system AcrIIA5‐LOV9, created by fusing the LOV9 domain consisting of AsLOV2 and cODC degron to the C‐terminus of AcrIIA5, which also showed obvious blue light‐dependent activity switch.

## RESULTS

### Construction and improvement of photo‐sensitive AcrIIA5

**Figure 1:**
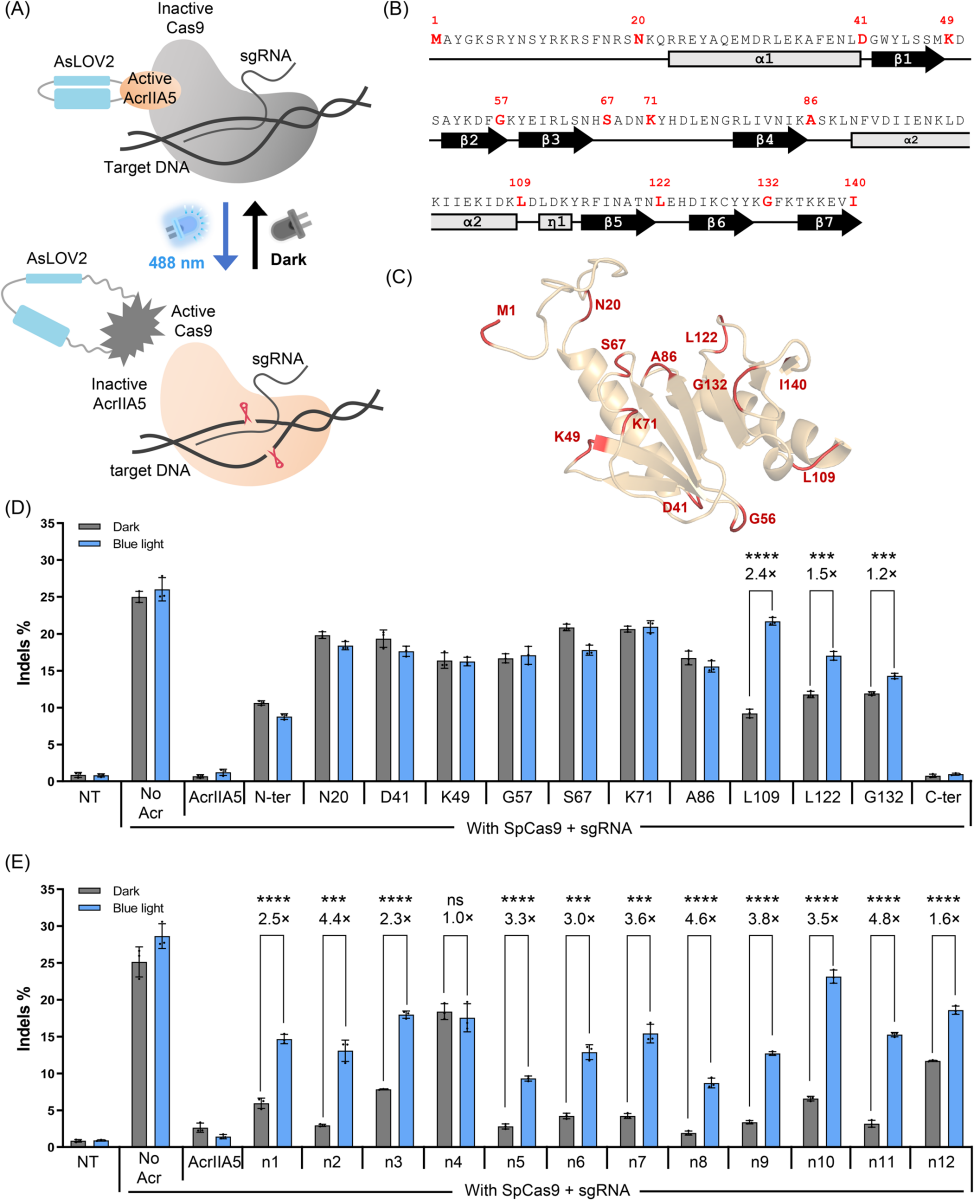
Construction and characterization of CASANOVA (CN)‐A5. (A) Schematic of CN‐A5. In the dark state, the unaltered AsLOV2 maintains the structural integrity of AcrIIA5, thereby preserving its inhibition of Cas9. Upon blue light irradiation, AsLOV2 undergoes a conformational change, disrupting the AcrIIA5 structure and restoring the inhibited Cas9 activity. (B) Display of the amino acid sequences and the secondary structure of AcrIIA5. Twelve candidate amino acid sites of AcrIIA5 for AsLOV2 insertion are highlighted in red and bold. (C) Schematic of AsLOV2 insertion sites in AcrIIA5. All candidate amino acid sites for AsLOV2 insertion are shown and red‐labeled. (D) Assessment of blue light‐dependent regulation of SpCas9 activity by different AcrIIA5–AsLOV2 hybrid variants. The plasmids expressing SpCas9, AcrIIA5–AsLOV2 hybrids, and the sgRNA targeting the *CCR5* locus were co‐transfected into HEK293T cells. The vector mole ratio of the AcrIIA5‐AsLOV2 hybrids:SpCas9 is 1:1. (E) Evaluation of blue light–dependent regulation of SpCas9 activity by newly designed CN‐A5 variants derived from the hybrid L109. The plasmids expressing SpCas9, the newly designed CN‐A5 variants, and the sgRNA targeting the *CCR5* locus were co‐transfected into HEK293T cells. The vector mole ratio of the AcrIIA5–AsLOV2 hybrids:SpCas9 is 1:1. At 12 h after transfection, the culture plate was placed below LED lamps for illumination, and the cells were cultured for an additional 48 h. Throughout the entire 60 h period, dark‐treated cells were wrapped in tinfoil. NT, not transfected. The fold change of indel formation is shown above the column. All data are shown as means ± SD for *n* = 3 biologically independent replicates. *p* values (***p < 0.001; ****p < 0.0001; ns, not significant) were calculated using two‐tailed Student's *t*‐test. CN‐A5, the hybrid L109.

To construct the photo‐sensitive AcrIIA5 hybrids (Figure 1A), we decided to choose the AsLOV2 domain from phototropin‐1 of *Avena sativa* as the blue light sensor and insert it into AcrIIA5^38^. In the dark state, the N‐terminal A'α and C‐terminal Jα helices of AsLOV2 are folded and tightly attached to the Per‐ARNT‐Sim (PAS) core. Thus, the structure of AcrIIA5 would remain intact, maintaining its inhibitory activity against Cas9 proteins, while upon blue light illumination, structural rearrangements occurred, leading to the unfolding of two terminal α‐helices^38^, ^39^. Such a substantial allostery of AsLOV2 further destroyed the structure of AcrIIA5, resulting in the loss of its inhibition activity. Consequently, the blocked cleavage activity of Cas9 would be restored.

Theoretically, the position at which the AsLOV2 was inserted would significantly impact the function of the AcrIIA5–AsLOV2 hybrid. As a reference, analysis of the complex structure of AcrIIA4 with SpCas9 (PDB code 5XBL) provided the guidance on the appropriate AsLOV2 insertion sites in the case of CASANOVA, a light‐responsive variant of AcrIIA4^31^. However, no complex structure between AcrIIA5 and any Cas9 protein has been reported. Thus, we investigated the nuclear magnetic resonance (NMR) structure of AcrIIA5 (PDB code 6LKF)^40^, showing three α‐helices and seven β‐sheets. Considering that the compact secondary structure would play an important role in activity retention, we selected at least one site in each unconsolidated loop of AcrIIA5 for subsequent insertion of AsLOV2.

To accurately measure the inhibition efficiency of AcrIIA5–AsLOV2 hybrids, SpCas9 was selected as the nuclease, and the *CCR5* gene, expressing the HIV receptor, was chosen as the target locus. Plasmids expressing AcrIIA5–AsLOV2 hybrids and SpCas9 were co‐transfected into HEK293T cells, which were then cultured under either dark conditions or blue light irradiation. The target locus was amplified and sequenced, and the insertions and deletions (indels) formation caused by nonhomologous end‐joining (NHEJ) was calculated using CRISPResso2^41^. Given that the cleavage activity of SpCas9 would show an inverse correlation with the inhibition capacity of AcrIIA5–AsLOV2 hybrids, we expected that the editing efficiency of SpCas9 with the appropriate photo‐sensitive AcrIIA5–AsLOV2 hybrid would approximate that of SpCas9 with wild‐type AcrIIA5 in the dark state and would approach that of SpCas9 alone under blue light irradiation.

The primary sequences and the secondary structure of AcrIIA5 are shown and specific insertion sites are marked (Figure 1B,C). The amino acid after which AsLOV2 is inserted represents the corresponding AcrIIA5–AsLOV2 hybrids. For example, the hybrid N20 indicates that AsLOV2 is inserted between N20 and K21. Notably, hybrids L109, L122 and G132 maintained fair inhibition ability to SpCas9 in the dark state and showed partial loss of inhibition capacity to SpCas9 after photoexcitation (Figures 1D and S1). We noticed that the three insertion sites were all located near the C‐terminus of AcrIIA5. Previous studies have indicated that the active residues of AcrIIA5 are located in the N‐terminal and the middle regions^40^, distant from the three suitable insertion sites L109, L122, and G132. As a result, these hybrids demonstrated considerable inhibition activity retained in the dark state.

We also observed that the hybrid L109 showed the most substantial loss of function under blue light, leading to a recovery in editing efficiency comparable to that of SpCas9 alone (Figure S2). After checking the structure of AcrIIA5, we found that the L109 was situated in the vicinity of the well‐structured connector η1 (Figure 1B,C), which was supposed to be a relatively rigid region. Hence, unfolding of the two terminal α‐helices in AsLOV2 under blue light was more likely to cause extensive disruption to the adjacent secondary structure of AcrIIA5, rendering it inactive finally. Then, the hybrid L109, named CN‐A5 (CASANOVA‐A5), was chosen to be further optimized to improve its performance.

Considering that the amino acid compositions at the boundaries of AsLOV2 and AcrIIA5 are crucial to the performance of hybrids, we subsequently fine‐tuned the amino acids surrounding L109 of AcrIIA5 through short deletions or insertions of flexible amino acids (Figure S3). Almost all newly designed CN‐A5 variants (hybrid n1–n12) showed substantial change in inhibition activity in the dark and blue light (Figures 1E and S4).

We found that short deletions were highly effective in enhancing the overall editing performance of CN‐A5 variants, whether in the dark state or under the blue light irradiation (e.g., CN‐A5‐n2), while some longer deletions resulted in complete inactivation (e.g., CN‐A5‐n4). Additionally, the variants with the insertion of flexible GS‐linkers (e.g., CN‐A5‐n5–n8) showed strong retention of inhibition activity against SpCas9 in the dark. We hypothesized that the flexible GS‐linker may alleviate the structural tension at the AcrIIA5–AsLOV2 interface, albeit at the expense of sensitivity to blue light, thereby enhancing the overall stability of AcrIIA5 (e.g., CN‐A5‐n5–n8). Therefore, it was not confusing to realize that the CN‐A5‐n10 with two amino acid deletions and a GSGG linker insertion showed particularly robust blue light‐switching behavior while maintaining significant inhibition activity against SpCas9 in the dark state (Figure S5).

**Figure 2:**
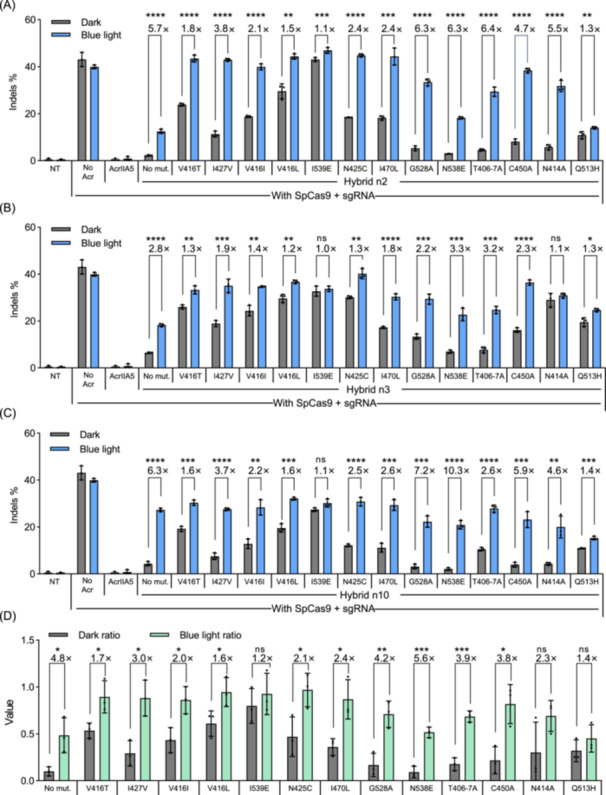
Optimization of insertion sites, linkers, and LOV2 mutations to enhance CN‐A5 performance. (A–C) Blue light control of mutated CN‐A5‐n2 (A), mutated CN‐A5‐n3 (B), and mutated CN‐A5‐n10 (C). The plasmids expressing SpCas9, CN‐A5 variants, and the sgRNA targeting the *CCR5* locus were co‐transfected into HEK293T cells. The vector mole ratio of the CN‐A5 variants:SpCas9 is 1:1. At 12 h after transfection, the culture plate was placed below LED lamps for illumination, and the cells were cultured for an additional 48 h. Throughout the entire 60 h period, dark‐treated cells were wrapped in tinfoil. No mut. means no mutation. All data are shown as means ± SD for *n* = 3 biologically independent replicates. (D) Comprehensive analysis of the effects of AsLOV2 point mutations on the light‐dependent regulatory activity of CN‐A5 variants. The “Dark ratio” was calculated using “indel formation efficiency of SpCas9 with CN‐A5 variants in the dark state” divided by “indel formation efficiency of SpCas9 without hybrids in the dark state.” The “Blue light ratio” was calculated using “indel formation efficiency of SpCas9 with CN‐A5 variants under blue light” divided by “indel formation efficiency of SpCas9 without hybrids under blue light.” The fold change is shown above the column. Each set of data was derived from the average indel formation rate from CN‐A5 variants n2, n3, and n10 and shown as means ± SD. *p* values (*p < 0.05; **p < 0.01; ***p < 0.001; ****p < 0.0001; ns, not significant) were calculated using two‐tailed Student's *t*‐test.

It has been reported that some amino acid point mutations surrounding the A'α and Jα helices of AsLOV2 could have a remarkable influence on its allosteric behavior^42^, ^43^, ^44^, ^45^, ^46^, ^47^, ^48^. We supposed that such engineering of AsLOV2 would directly affect the performance of CN‐A5. Therefore, we introduced 13 amino acid mutations into CN‐A5 variants n2, n3, and n10, aiming to create the superior photo‐sensitive AcrIIA5 (Figure S6). We noticed that all mutations caused varying degrees of changes in the editing performance compared to the original hybrids (Figures 2A–C and S7).

We conducted a comprehensive analysis of the effects of these mutations on all 42 CN‐A5 variants and quantified them using three indicators. In the “Dark ratio” index (indel formation efficiency of SpCas9 with CN‐A5 variants/indel formation efficiency of SpCas9 in the dark), some mutations, V416T, V416I, V416L, and I539E, resulted in high background editing. Conversely, the variants with mutations G528A, N538E, T406‐7A, and C450A maintained lower background editing, only slightly higher than those with no mutation (Figure 2D). Regarding the “Blue light ratio” index (indel formation efficiency of SpCas9 with CN‐A5 variants/indel formation efficiency of SpCas9 under blue light), all hybrids reached the value of at least 0.6, except those with the mutations N538E and Q513H, which indicated low sensitivity to blue light (Figure 2D). Furthermore, the fold change between the “Dark ratio” and the “Blue light ratio” represents the dynamic range of hybrids to some extent. We found that the hybrids with G528A, N538E, T406‐7A, and C450A showed satisfactory fold changes, comparable to or better than those of the hybrids with no mutations.

It has been reported that mutations near the flavin mononucleotide (FMN)‐binding pocket could affect the photocycle lifetime of AsLOV2^42^, ^43^, ^44^, ^47^, ^48^. According to previous research, mutations such as V416T, I427V, N425C, I470L, and Q513H would shorten the photocycle of AsLOV2, while mutations like V416I, V416L, and N414A would increase it (Table S1). From the analysis, it was evident that all these mutations altered the dynamic range of CN‐A5 variants, and it appeared that shortening the photocycle would be more beneficial in improving their performance.

Another group of mutations was located in the N‐terminal A'α and the C‐terminal Jα helices of AsLOV2^45^, ^48^. The T406‐7A double mutations aiming at stabilizing the N‐terminal A'α helix of AsLOV2 and enhancing Jα docking affinity resulted in improvements in the dynamic range of CN‐A5 variants, particularly for the variant CN‐A5‐n2 (Figure 2A–C). Similarly, mutations such as G528A and N538E were designed to stabilize the interaction between the Jα helix and the PAS core domain in both the dark and lit states, resulting in significant enhancements to the dynamic range of CN‐A5 variants (Figure 2D).

Moreover, the mutation C450A, anticipated to maintain AsLOV2 in the dark state continuously and deactivate the photocycle through disrupting the covalent formation between C450 and FMN in AsLOV2, still remained light‐responsive and even improved the performance of hybrids considerably (Figure 2D). Conversely, the mutation I539E, which was reported to disrupt the Jα helix−PAS core domain interaction and lock AsLOV2 in the lit state, led to a markedly reduced dynamic range in the tested variants (Figure 2D).

### CN‐A5 and its variants show broad spectrum

The above results indicated that the CN‐A5 variants n2‐G528A, n2‐T406‐7A, n2‐C450A, and n10 could induce significant release of SpCas9's activity under blue light exposure. Considering that AcrIIA5 showed broad inhibition toward other type II Cas9 orthologs, we hypothesized that the photo‐sensitive CN‐A5 could also modulate the activity of other smaller‐sized Cas9 orthologs such as SaCas9, NmeCas9, and St1Cas9.

Similar to the case of SpCas9, plasmids expressing Cas9 orthologs, sgRNA, and CN‐A5 variants were co‐transfected into HEK293T cells. Meanwhile, different mole ratios of CN‐A5:Cas9 were tested. Surprisingly, the results revealed that various CN‐A5 variants showed distinct regulatory mechanisms on different Cas9 proteins.

**Figure 3:**
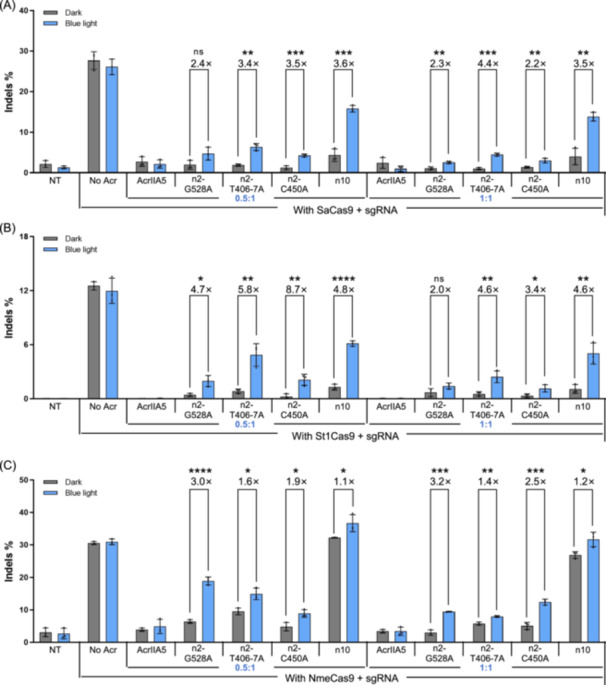
Examination of the regulation ability of CN‐A5 variants on Cas9 orthologs. (A–C) Blue light control of CN‐A5 variants n2‐G528A, n2‐T406‐7A, n2‐C450A, and n10 to SaCas9 (A), St1Cas9 (B), and NmeCas9 (C). The plasmids expressing Cas9 orthologs, CN‐A5 variants, and sgRNA were co‐transfected into HEK293T cells. The vector mole ratio of the CN‐A5 variants:Cas9 orthologs is indicated in blue‐marked numbers below the x‐coordinate. At 12 h after transfection, the culture plate was placed below LED lamps for illumination, and the cells were cultured for an additional 48 h. Throughout the entire 60 h period, dark‐treated cells were wrapped in tinfoil. All data are shown as means ± SD for *n* = 3 biologically independent replicates. *p* values (*p < 0.05; **p < 0.01; ***p < 0.001; ****p < 0.0001; ns, not significant) were calculated using two‐tailed Student's *t*‐test.

SaCas9 with CN‐A5‐n10 at a dose of a 1:0.5 mole ratio showed relatively low background editing of 4.3% in the dark, which was 15.7% of that observed in the group of SaCas9 alone. Upon blue light irradiation, a significant increase in indel formation efficiency was observed, reaching 15.8%, which was approximately 60.6% of SaCas9 alone. We also noticed that increased CN‐A5:Cas9 vector ratios efficiently reduced background editing in the dark, albeit at the cost of some loss of Cas9 activity upon irradiation. Furthermore, we observed that variants CN‐A5‐n2‐G528A, T406‐7A, and C450A showed effective changes in inhibition ability in the presence and absence of blue light, but the blue light‐dependent editing was relatively limited (Figures 3A and S8A).

Similar to SaCas9, St1Cas9 with CN‐A5‐n10 at a dose of a 1:0.5 mole ratio demonstrated a blue light‐dependent editing efficiency of 6.1% and a background editing efficiency of 1.3% in the dark. Likewise, the variants CN‐A5‐n2‐G528A, T406‐7A, and C450A also showed poor performance in regulating the editing of St1Cas9 (Figures 3B and S8B).

However, unlike in the case of SaCas9 and St1Cas9, CN‐A5‐n10 had almost no inhibitory effect on NmeCas9, and no apparent change was observed in the presence and absence of blue light. Nonetheless, NmeCas9 with CN‐A5‐n2‐G528A at a 1:0.5 mole ratio showed a high blue light‐dependent editing efficiency of 18.9%, which reached 61.2% of NmeCas9 alone, and low background editing efficiency of 6.4% in the dark, which was 20.9% of NmeCas9 alone (Figures 3C and S8C).

Overall, we have confirmed that the developed CN‐A5 variants showed good regulatory effects on multiple Cas9 orthologs. However, their efficacy was lower than that observed for SpCas9, with CN‐A5‐n10 exhibiting only limited regulation of NmeCas9. We hypothesized that the limited universality of CN‐A5 may be attributed to the underlying differences in the mechanism by which AcrIIA5 inhibits various Cas9 orthologs. Specifically, AcrIIA5 may utilize different regions to bind and suppress different Cas9 orthologs, as suggested by the finding that a theoretically inactive mutant of AcrIIA5, AcrIIA5H_66_N_70_H_73_ ^36^, is unable to inhibit NmeCas9 but still effectively inhibits SpCas9. Building on this, we observed that SpCas9, SaCas9, and St1Cas9, all belonging to the type II‐A subfamily, were efficiently regulated by CN‐A5‐n10 (Figure 3). In contrast, NmeCas9, a type II‐C Cas9, showed only limited regulation by CN‐A5‐n10, suggesting that differences between Cas9 subfamilies primarily determined the performance of light‐sensitive Acrs. Furthermore, the regulatory capability of CN‐A5 variants can vary even within the same Cas9 subfamily, as demonstrated by the differing effects of CN‐A5‐n2‐G528A on and St1Cas9. This underscored the importance of evaluating multiple CN‐A5 variants for a specific Cas9 to fully understand their regulatory potential. Of course, the CN‐A5‐n10 was the most applicable one, which should be tested first.

Apart from efficiency, safety is a crucial consideration for potential applications. Therefore, we further investigated whether these CN‐A5 variants could influence the off‐target effects of the Cas9 orthologs. The results indicated that none of the tested loci showed off‐target editing, regardless of the presence or absence of the photo‐sensitive AcrIIA5 hybrids under blue light, suggesting that CN‐A5 may offer a high level of safety, as no additional off‐target editing was observed compared to Cas9 alone (Figure S9).

### Construction and improvement of the degron‐based photo‐sensitive AcrIIA5

**Figure 4:**
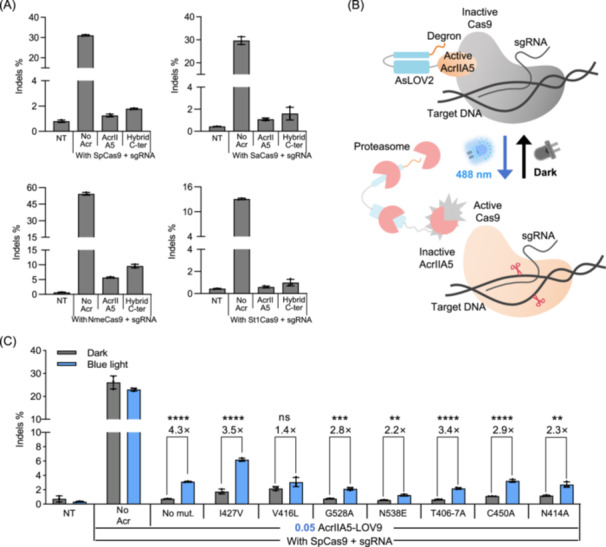
Construction and improvement of degron‐based photo‐sensitive AcrIIA5. (A) Comparison of the inhibition ability of AcrIIA5 and hybrid C‐ter with SpCas9, SaCas9, NmeCas9, and St1Cas9. The plasmids expressing Cas9 orthologs, AcrIIA5–AsLOV2 hybrids, and sgRNA were co‐transfected into HEK293T cells. The mole ratio of AcrIIA5–AsLOV2 hybrids:Cas9 was 0.1:1. The transfected cells were wrapped in tinfoil and cultured for 60 h. (B) Schematic of AcrIIA5‐LOV9 (AsLOV2 + Degron). In the dark state, the interaction between the Jα helix and the Per‐ARNT‐Sim (PAS) core effectively stabilizes the degron, preventing its release. Upon blue light irradiation, the Jα helix of AsLOV2 undergoes unfolding, which disrupts the binding and allows the degron to be recognized by the ubiquitin‐independent proteasome, leading to the degradation of the fused AcrIIA5 and the restoration of the inhibited Cas9 activity. (C) Mutational enhancement of light‐dependent degradation efficiency for AcrIIA5‐LOV9 variants. The plasmids expressing SpCas9, AcrIIA5‐LOV9 variants, and the sgRNA targeting the *CCR5* locus were co‐transfected into HEK293T cells. The mole ratio of AcrIIA5‐LOV9:Cas9 is indicated in blue number. At 12 h after transfection, the culture plate was placed below LED lamps for illumination, and the cells were cultured for an additional 48 h. Throughout the entire 60 h period, dark‐treated cells were wrapped in tinfoil. All data are shown as means ± SD for *n* = 3 biologically independent replicates. *p* values (*p < 0.05; **p < 0.01; ***p < 0.001; ****p < 0.0001; ns, not significant) were calculated using two‐tailed Student's *t*‐test.

We observed that when AsLOV2 was fused to the C‐terminus of AcrIIA5, it lost the responsiveness to blue light and showed similar inhibitory effect on SpCas9 compared to the wild‐type AcrIIA5 (Figure 1D). We speculated that under blue light irradiation, the conformational changes induced by the movement of the Jα helix, which were then transmitted through the PAS scaffold to the active site of AcrIIA5, became negligible. We tested the indel formation efficiency of SpCas9, SaCas9, NmeCas9, and St1Cas9 with either AcrIIA5 or hybrid C‐ter at various concentrations (Figures 4A and S10). The results indicated that even at very low molar ratios, hybrid C‐ter still maintained a robust and stable inhibitory effect, suggesting that it was a potent variant similar to the wild‐type AcrIIA5.

Recently, a light‐sensitive degron system LOV9 was constructed by attaching a small degron cODC to the C‐terminus of AsLOV2^49^, ^50^. In the dark state, the constraint caused by the docking of Jα helix to PAS core could hold the degron tightly. Upon blue light illumination, unfolding of the Jα helix of AsLOV2 would release the locking of the degron, which was in turn recognized by the ubiquitin‐independent proteasome and then mediated degradation of the fused protein.

Since both the hybrid C‐ter and LOV9 (AsLOV2 + Degron) shared the same blue light sensor AsLOV2, we constructed AcrIIA5‐LOV9 by fusing the LOV9 domain to the C‐terminus of AcrIIA5 (Figure 4B). In the dark state, the caged degron could not be recognized by proteasome, allowing AcrIIA5 to remain active, while under photoexcitation, released cODC degron ultimately triggered the degradation of AcrIIA5 by the proteasome.

Meanwhile, we introduced mutations I427V, V416L, G528A, N538E, T406‐7A, C450A, and N414A, which have been proven to be useful in modulating the activity of AsLOV2, into AcrIIA5‐LOV9. Subsequently, we investigated the blue light‐dependent regulation of SpCas9 activity at three dose ratios of AcrIIA5‐LOV9 and its variants. The results indicated that AcrIIA5‐LOV9 and its point mutation variants at all three concentrations showed indel formation close to background levels in the dark and demonstrated observable blue light‐dependent gene editing efficiency (Figures 4C and S11). Among them, the AcrIIA5‐LOV9‐I427V at a concentration of a 0.05:1 mole ratio showed considerable blue light‐dependent activity change of 3.5‐fold. However, we found that the indel formation efficiency of AcrIIA5‐LOV9‐I427V, even the best variant, was a quarter of the SpCas9 alone under the blue light, indicating that further engineering should be carried out to improve its performance. Additionally, we observed that AcrIIA5‐LOV9 with V416L almost completely eliminated the sensitivity to blue light. These results indicated that a photocycle might be more important than the docking affinity of Jα to the PAS core in this degron‐based AcrIIA5 system compared with the case of CN‐A5.

Taken together, we have proven that the degron‐based photo‐sensitive AcrIIA5 system could efficiently regulate the cleavage activity of SpCas9. Although we have not tested the AcrIIA5‐LOV9 toward other Cas9 orthologs, we have affirmed that AcrIIA5 can effectively inhibit the activity of SaCas9, NmeCas9, and St1Cas9 at low concentrations (Figure S10). Considering that the LOV9 domain is fused to the C‐terminus of AcrIIA5, which is not the crucial region for binding with Cas9 (Figure 1E), we expect that AcrIIA5‐LOV9 would retain its broad inhibition spectrum. Therefore, AcrIIA5‐LOV9 is likely to be applicable to other Cas9 orthologs as well.

## DISCUSSION

The application of CRISPR‐Cas9‐based technology has greatly facilitated gene manipulation. However, concerns have been raised about the potential for severe genetic toxicity associated with double‐strand breaks induced by CRISPR‐Cas9^51^. Recently, the appearance of Cas9 variants with improved HDR (homology‐directed repair) efficiency has reignited interest in this method^52^, ^53^. Besides, there was evidence that optogenetic regulation of Cas9 will enable improvement in its safety, which was considered as one of the most important factors in precise gene therapy^34^.

In this study, we incorporated AsLOV2 into AcrIIA5, yielding a series of CN–A5 hybrids. These blue light‐switchable AcrIIA5 hybrids showed robust inhibition to the cleavage of various Cas9 proteins, including SpCas9, SaCas9, NmeCas9, and St1Cas9, in the absence of light irradiation, while in the presence of blue light, these photo‐sensitive CN‐A5 released the activity of Cas9, resulting in considerable editing efficiency. Moreover, the investigation of the off‐target effects indicated that the participation of CN‐A5 may maintain or improve the safety of these Cas9 proteins. In addition to considering the insertion site and linker design when engineering AcrIIA5–AsLOV2 variants, we also explored the effects of mutations intended to modulate the AsLOV2 photocycle. Among these mutations, C450A and I539E yielded particularly interesting results. Theoretically, the two mutations were expected to render the CN‐A5 variants insensitive, and yet, the outcome was nearly the opposite. This opposite phenomenon suggested that the practical operational process of the CN‐A5 would likely be more intricate. One possible explanation is that the AsLOV2‐C450A can carry out conformational change in a covalent‐independent manner, which was observed less in wild‐type AsLOV2^54^, ^55^. This alternative recycle of FMN made those AcrIIA5–AsLOV2 hybrids with the C450A mutation in AsLOV2 function in the dark state and light state, which cannot be applied to those hybrids with I539E.

Based on the aforementioned findings, we can draw a conclusion that altering the affinity of the N‐terminal A'α and C‐terminal Jα helices to the PAS core domain would enhance the performance of the CN‐A5, compared to modifying the photocycle. A possible explanation is that variations that shorten or prolong the photocycle often also negatively impact on signal transduction, thereby decreasing the performance of the AcrIIA5–AsLOV2 hybrids with mutations to change the photocycle. This insight could prove beneficial in the engineering of other types of Acr‐based optogenetic tools. Furthermore, it is worth noting that different CN‐A5 variants may show inconsistent changes in editing performance when mutations are introduced (Figure 2A–C). Hence, incorporating mutations into more CN‐A5 variants would help in identifying the superior one.

In parallel, we also developed the cODC degron‐based AcrIIA5‐LOV9, which showed observable blue light‐dependent regulation for SpCas9. However, although AcrIIA5‐LOV9 maintained strong inhibition of SpCas9 in the dark, it was unable to release sufficient SpCas9 cleavage activity after illumination. We hypothesized that this might be due to the degron in the dissociated‐Jα helix not having enough time to recruit the proteasome after blue light stimulation. Hence, the AcrIIA5‐LOV9‐I427V variant with a changed photocycle finally improved the light‐dependent activity of SpCas9. We speculated that the overall performance might be further improved by introducing additional point mutations related to photocycle change in AsLOV2. Besides, replacing the cODC degron with other degrons that have higher activity could further increase the sensitivity of AcrIIA5‐LOV9.

Apart from SpCas9, SaCas9, NmeCas9, and St1Cas9, AcrIIA5 has the potential to inhibit other Type II Cas9 orthologs such as HpaCas9, GeoCas9, and CjeCas9^36^. Therefore, it is conceivable that these Cas9 orthologs could also be regulated by CN‐A5 and AcrIIA5‐LOV9, expanding the application of the photo‐sensitive AcrIIA5.

Moreover, our study identified several point mutations that could enhance the sensitivity of CN‐A5 to blue light. We believe that these point mutations may be applied to other blue light‐switchable Acrs^33^, serving as a general modification strategy.

Despite these promising results, we acknowledge certain limitations in the current study. Specifically, the off‐target profiling of St1Cas9 was performed at a relatively modest sequencing depth. As a result, the sensitivity for detecting low‐frequency off‐target events may be compromised, which in turn could limit the statistical robustness of downstream analyses. To address this issue, future investigations employing deeper sequencing coverage or orthogonal validation methods (such as GUIDE‐seq) will be helpful for further refining the off‐target landscape of CN‐A5‐regulated Cas9 variants.

In summary, we engineered a series of blue‐light‐responsive AcrIIA5 proteins by combining the broad‐spectrum Acr protein AcrIIA5 with the light‐sensitive protein AsLOV2 through domain insertion and degron strategies. Additionally, we outlined specific optimization approaches that could be applied to modify other photosensitive Acr proteins.

## MATERIALS AND METHODS

### Plasmid cloning

Cloning was carried out using the Hieff Clone One Step Cloning Kit (YEASEN). The DNA fragments of *AcrIIA5*, *AsLOV2*, sgRNA scaffolds of *SpCas9*, *SaCas9*, *NmeCas9*, and *St1Cas9* were synthesized by GenScript. PCR was performed using Phanta Max Super‐Fidelity DNA polymerase (Vazyme). Site‐directed mutagenesis of *AsLOV2* was performed using Mut Express II Fast Mutagenesis Kit V2 (Vazyme).

The DNA fragments of *SpCas9*, *SaCas9*, *NmeCas9*, and *St1Cas9* were amplified from Px459 V2.0 (Addgene #62988), BPK2101 (Addgene #65770), pEJS654 (Addgene #112139), and MSP1673 (Addgene #65769), respectively. These fragments were cloned into the backbone of pCMV‐BE3 (Addgene #73021) to construct pCMV‐SpCas9, pCMV‐SaCas9, pCMV‐NmeCas9, and pCMV‐St1Cas9. The DNA fragment of *AcrIIA5* was amplified and cloned into the backbone of pcDNA3.1(‐) to construct pCMV‐AcrIIA5. The AcrIIA5–AsLOV2 hybrids were constructed by Golden Gate assembly. The sgRNA scaffolds of *SpCas9*, *SaCas9*, *NmeCas9*, and *St1Cas9* were cloned into pUC57 under the U6 promoter amplified from the PX459 v2.0 plasmid. Protospacer oligos were phosphorylated by PNK (Thermo Fisher Scientific) and cloned into sgRNA expression plasmids by T4 DNA Ligase (Thermo Fisher Scientific). The amino acid sequences or nucleotide sequences are listed in Supplementary Sequence 1 and Supplementary Sequence 2 in Supporting Information section.

### Setup for blue light irradiation

Six LED lamps with a peak wavelength of 465 nm were used for blue light illumination. The intensity of irradiation was assessed with a light meter (A5813, SMART SENSOR) and fine‐tuned by altering the distance between the lamps and the plates. The average light intensity for HEK293T cells was 2 mW cm^−2^. For the dark group, the plates were enclosed in tinfoil.

### Culture condition and transfection

HEK293T (ATCC CRL‐3216) cells were maintained in a growth medium consisting of Dulbecco's Modified Eagle Medium (DMEM, Gibco) with 10% fetal bovine serum (FBS, Gibco) and cultured under conditions of 37°C and 5% CO_2_.

HEK293T cells were plated in 48‐well plates (Corning) at a density of 55,000 cells per well and transfected 18 h later with Lipofectamine 3000 (Invitrogen) according to the manufacturer's protocol. 500 ng plasmids expressing pCMV‐SpCas9, pCMV‐SaCas9, pCMV‐NmeCas9, or pCMV‐St1Cas9 were transfected with 200 ng of corresponding sgRNA‐expressing plasmids. When measuring the blue light‐controlled indel formation by Cas9, plasmids expressing AcrIIA5 or AcrIIA5–AsLOV2 hybrids were co‐transfected at various doses, as indicated in the figure legends. At 12 h after transfection, the culture plate was placed below six LED lamps (2 mW cm^−2^; 465 nm) for illumination, and the cells were cultured for an additional 48 h. Throughout the entire 60 h period, dark‐treated cells were wrapped in tinfoil.

### Extraction of genomic DNA

Genomic DNA from HEK293T cells was extracted by washing the cells with PBS and lysing them in 100 μL of lysis buffer (10 mM Tris‐HCl, pH 8.0, 5 mM EDTA, 0.1% SDS, and 30 μg ml^−1^ proteinase K (YEASEN)) at 37°C for 1 h. The lysate was then heated at 80°C for 20 min, and the resulting DNA solution was stored at –80°C.

### Amplicon sequencing

The primers used to amplify the target regions are listed in Table S2. 50 µl PCR reactions were purified using the TIANquick Maxi Purification Kit (TIANGEN). Illumina‐compatible adapters and indices were added to the PCR products. Final amplicon libraries were sequenced with 150 bp paired‐end reads on an Illumina MiSeq instrument, and the RAW data were analyzed using the CRISPRosso2^41^. The “Indel_histogram” file was used to calculate the indel formation. The sgRNA‐dependent off‐target sites were investigated using Cas‐OFFinder^56^. The primers used to amplify the off‐target regions are listed in Table S3.

### Statistics analysis

All statistical analyses were performed on at least three biologically independent experiments using an unpaired two‐tailed Student's *t*‐test through GraphPad Prism 9 (GraphPad Software).

## AUTHOR CONTRIBUTIONS

**Qi Chen:** Conceptualization; data curation; formal analysis; writing—original draft; writing—review and editing. **Jia Yao:** Data curation; formal analysis. **Yingfan Lu:** Data curation; formal analysis. **Ruikang Qiu:** Data curation. **Zixin Deng:** Supervision. **Yuhui Sun:** Conceptualization; formal analysis; funding acquisition; project administration; supervision; writing—original draft; writing—review and editing.

## ETHICS STATEMENT

The study in this article did not involve any trials on humans or animals.

## CONFLICT OF INTERESTS

The authors declare no conflict of interests.

## Supporting information

Revised Supporting Information.

Supporting Data.

## Data Availability

HTS data have been deposited in the National Center for Biotechnology Information (NCBI) Sequence Read Archive (SRA) database under the accession codes PRJNA1116865 and PRJNA1117012.

## References

[1] Wiedenheft B , Sternberg SH , Doudna JA . RNA‐guided genetic silencing systems in bacteria and archaea. Nature. 2012;482:331–338. 22337052 10.1038/nature10886

[2] Jinek M , Chylinski K , Fonfara I , Hauer M , Doudna JA , Charpentier E . A programmable dual‐RNA‐guided DNA endonuclease in adaptive bacterial immunity. Science. 2012;337:816–821. 22745249 10.1126/science.1225829 PMC6286148

[3] Cong L , Ran FA , Cox D , Lin S , Barretto R , Habib N , et al. Multiplex genome engineering using CRISPR/Cas systems. Science. 2013;339:819–823. 23287718 10.1126/science.1231143 PMC3795411

[4] Mali P , Yang L , Esvelt KM , Aach J , Guell M , DiCarlo JE , et al. RNA‐guided human genome engineering via Cas9. Science. 2013;339:823–826. 23287722 10.1126/science.1232033 PMC3712628

[5] Dever DP , Bak RO , Reinisch A , Camarena J , Washington G , Nicolas CE , et al. CRISPR/Cas9 β‐globin gene targeting in human haematopoietic stem cells. Nature. 2016;539:384–389. 27820943 10.1038/nature20134 PMC5898607

[6] Santiago‐Fernández O , Osorio FG , Quesada V , Rodríguez F , Basso S , Maeso D , et al. Development of a CRISPR/Cas9‐based therapy for Hutchinson‐Gilford progeria syndrome. Nat Med. 2019;25:423–426. 30778239 10.1038/s41591-018-0338-6 PMC6546610

[7] Frangoul H , Altshuler D , Cappellini MD , Chen YS , Domm J , Eustace BK , et al. CRISPR‐Cas9 gene editing for sickle cell disease and β‐thalassemia. N Engl J Med. 2021;384:252–260. 33283989 10.1056/NEJMoa2031054

[8] Nuñez JK , Chen J , Pommier GC , Cogan JZ , Replogle JM , Adriaens C , et al. Genome‐wide programmable transcriptional memory by CRISPR‐based epigenome editing. Cell. 2021;184:2503–2519.e17. 33838111 10.1016/j.cell.2021.03.025 PMC8376083

[9] Kearns NA , Pham H , Tabak B , Genga RM , Silverstein NJ , Garber M , et al. Functional annotation of native enhancers with a Cas9‐histone demethylase fusion. Nat Methods. 2015;12:401–403. 25775043 10.1038/nmeth.3325 PMC4414811

[10] Hilton IB , D'Ippolito AM , Vockley CM , Thakore PI , Crawford GE , Reddy TE , et al. Epigenome editing by a CRISPR‐Cas9‐based acetyltransferase activates genes from promoters and enhancers. Nat Biotechnol. 2015;33:510–517. 25849900 10.1038/nbt.3199 PMC4430400

[11] Mali P , Aach J , Stranges PB , Esvelt KM , Moosburner M , Kosuri S , et al. Cas9 transcriptional activators for target specificity screening and paired nickases for cooperative genome engineering. Nat Biotechnol. 2013;31:833–838. 23907171 10.1038/nbt.2675 PMC3818127

[12] Bikard D , Jiang W , Samai P , Hochschild A , Zhang F , Marraffini LA . Programmable repression and activation of bacterial gene expression using an engineered CRISPR‐Cas system. Nucleic Acids Res. 2013;41:7429–7437. 23761437 10.1093/nar/gkt520 PMC3753641

[13] Gilbert LA , Horlbeck MA , Adamson B , Villalta JE , Chen Y , Whitehead EH , et al. Genome‐scale CRISPR‐mediated control of gene repression and activation. Cell. 2014;159:647–661. 25307932 10.1016/j.cell.2014.09.029 PMC4253859

[14] Ran FA , Cong L , Yan WX , Scott DA , Gootenberg JS , Kriz AJ , et al. In vivo genome editing using *Staphylococcus aureus* Cas9. Nature. 2015;520:186–191. 25830891 10.1038/nature14299 PMC4393360

[15] Esvelt KM , Mali P , Braff JL , Moosburner M , Yaung SJ , Church GM . Orthogonal Cas9 proteins for RNA‐guided gene regulation and editing. Nat Methods. 2013;10:1116–1121. 24076762 10.1038/nmeth.2681 PMC3844869

[16] Kleinstiver BP , Prew MS , Tsai SQ , Topkar VV , Nguyen NT , Zheng Z , et al. Engineered CRISPR‐Cas9 nucleases with altered PAM specificities. Nature. 2015;523:481–485. 26098369 10.1038/nature14592 PMC4540238

[17] Ibraheim R , Song CQ , Mir A , Amrani N , Xue W , Sontheimer EJ . All‐in‐one adeno‐associated virus delivery and genome editing by *Neisseria meningitidis* Cas9 in vivo. Genome Biol. 2018;19:137. 30231914 10.1186/s13059-018-1515-0 PMC6146650

[18] Agudelo D , Carter S , Velimirovic M , Duringer A , Rivest JF , Levesque S , et al. Versatile and robust genome editing with *Streptococcus thermophilus* CRISPR1‐Cas9. Genome Res. 2020;30:107–117. 31900288 10.1101/gr.255414.119 PMC6961573

[19] Pattanayak V , Lin S , Guilinger JP , Ma E , Doudna JA , Liu DR . High‐throughput profiling of off‐target DNA cleavage reveals RNA‐programmed Cas9 nuclease specificity. Nat Biotechnol. 2013;31:839–843. 23934178 10.1038/nbt.2673 PMC3782611

[20] Tsai SQ , Zheng Z , Nguyen NT , Liebers M , Topkar VV , Thapar V , et al. GUIDE‐seq enables genome‐wide profiling of off‐target cleavage by CRISPR‐Cas nucleases. Nat Biotechnol. 2015;33:187–197. 25513782 10.1038/nbt.3117 PMC4320685

[21] Zetsche B , Volz SE , Zhang F . A split‐Cas9 architecture for inducible genome editing and transcription modulation. Nat Biotechnol. 2015;33:139–142. 25643054 10.1038/nbt.3149 PMC4503468

[22] Dow LE , Fisher J , O'Rourke KP , Muley A , Kastenhuber ER , Livshits G , et al. Inducible in vivo genome editing with CRISPR‐Cas9. Nat Biotechnol. 2015;33:390–394. 25690852 10.1038/nbt.3155 PMC4390466

[23] Davis KM , Pattanayak V , Thompson DB , Zuris JA , Liu DR . Small molecule‐triggered Cas9 protein with improved genome‐editing specificity. Nat Chem Biol. 2015;11:316–318. 25848930 10.1038/nchembio.1793 PMC4402137

[24] Ermak G , Cancasci VJ , Davies KJA . Cytotoxic effect of doxycycline and its implications for tet‐on gene expression systems. Anal Biochem. 2003;318:152–154. 12782044 10.1016/s0003-2697(03)00166-0

[25] Laplante M , Sabatini DM . mTOR signaling in growth control and disease. Cell. 2012;149:274–293. 22500797 10.1016/j.cell.2012.03.017 PMC3331679

[26] Bursch W , Ellinger A , Kienzl H , Török L , Pandey S , Sikorska M , et al. Active cell death induced by the anti‐estrogens tamoxifen and ICI 164 384 in human mammary carcinoma cells (MCF‐7) in culture: the role of autophagy. Carcinogenesis. 1996;17:1595–1607. 8761415 10.1093/carcin/17.8.1595

[27] Nihongaki Y , Kawano F , Nakajima T , Sato M . Photoactivatable CRISPR‐Cas9 for optogenetic genome editing. Nat Biotechnol. 2015;33:755–760. 26076431 10.1038/nbt.3245

[28] Richter F , Fonfara I , Bouazza B , Schumacher CH , Bratovič M , Charpentier E , et al. Engineering of temperature‐ and light‐switchable Cas9 variants. Nucleic Acids Res. 2016;44:10003–10014. 27744350 10.1093/nar/gkw930 PMC5175372

[29] Hemphill J , Borchardt EK , Brown K , Asokan A , Deiters A . Optical control of CRISPR/Cas9 gene editing. J Am Chem Soc. 2015;137:5642–5645. 25905628 10.1021/ja512664v PMC4919123

[30] Yu Y , Wu X , Guan N , Shao J , Li H , Chen Y , et al. Engineering a far‐red light‐activated split‐Cas9 system for remote‐controlled genome editing of internal organs and tumors. Sci Adv. 2020;6:eabb1777. 32923591 10.1126/sciadv.abb1777 PMC7455487

[31] Bubeck F , Hoffmann MD , Harteveld Z , Aschenbrenner S , Bietz A , Waldhauer MC , et al. Engineered anti‐CRISPR proteins for optogenetic control of CRISPR‐Cas9. Nat Methods. 2018;15:924–927. 30377362 10.1038/s41592-018-0178-9

[32] He L , Tan P , Zhu L , Huang K , Nguyen NT , Wang R , et al. Circularly permuted LOV2 as a modular photoswitch for optogenetic engineering. Nat Chem Biol. 2021;17:915–923. 33958793 10.1038/s41589-021-00792-9

[33] Song G , Tian C , Li J , Zhang F , Peng Y , Gao X , et al. Rapid characterization of anti‐CRISPR proteins and optogenetically engineered variants using a versatile plasmid interference system. Nucleic Acids Res. 2023;51:12381–12396. 37930830 10.1093/nar/gkad995 PMC10711425

[34] Hoffmann MD , Mathony J , Upmeier zu Belzen J , Harteveld Z , Aschenbrenner S , Stengl C , et al. Optogenetic control of *Neisseria meningitidis* Cas9 genome editing using an engineered, light‐switchable anti‐CRISPR protein. Nucleic Acids Res. 2021;49:e29. 33330940 10.1093/nar/gkaa1198 PMC7969004

[35] Hynes AP , Rousseau GM , Agudelo D , Goulet A , Amigues B , Loehr J , et al. Widespread anti‐CRISPR proteins in virulent bacteriophages inhibit a range of Cas9 proteins. Nat Commun. 2018;9:2919. 30046034 10.1038/s41467-018-05092-w PMC6060171

[36] Garcia B , Lee J , Edraki A , Hidalgo‐Reyes Y , Erwood S , Mir A , et al. Anti‐CRISPR AcrIIA5 potently inhibits all Cas9 homologs used for genome editing. Cell Rep. 2019;29:1739–1746.e5. 31722192 10.1016/j.celrep.2019.10.017 PMC6910239

[37] Song G , Zhang F , Zhang X , Gao X , Zhu X , Fan D , et al. AcrIIA5 inhibits a broad range of Cas9 orthologs by preventing DNA target cleavage. Cell Rep. 2019;29:2579–2589.e4. 31775029 10.1016/j.celrep.2019.10.078

[38] Harper SM , Neil LC , Gardner KH . Structural basis of a phototropin light switch. Science. 2003;301:1541–1544. 12970567 10.1126/science.1086810

[39] Halavaty AS , Moffat K . N‐ and C‐terminal flanking regions modulate light‐induced signal transduction in the LOV2 domain of the blue light sensor phototropin 1 from *Avena sativa* . Biochemistry. 2007;46:14001–14009. 18001137 10.1021/bi701543e

[40] SY An , Ka D , Kim I , Kim EH , Kim NK , Bae E , et al. Intrinsic disorder is essential for Cas9 inhibition of anti‐CRISPR AcrIIA5. Nucleic Acids Res. 2020;48:7584–7594. 32544231 10.1093/nar/gkaa512 PMC7367191

[41] Clement K , Rees H , Canver MC , Gehrke JM , Farouni R , Hsu JY , et al. CRISPResso2 provides accurate and rapid genome editing sequence analysis. Nat Biotechnol. 2019;37:224–226. 30809026 10.1038/s41587-019-0032-3 PMC6533916

[42] Kay CWM , Schleicher E , Kuppig A , Hofner H , Rüdiger W , Schleicher M , et al. Blue light perception in plants. J Biol Chem. 2003;278:10973–10982. 12525505 10.1074/jbc.M205509200

[43] Harper SM , Christie JM , Gardner KH . Disruption of the LOV−Jα helix interaction activates phototropin kinase activity. Biochemistry. 2004;43:16184–16192. 15610012 10.1021/bi048092i

[44] Wang H , Vilela M , Winkler A , Tarnawski M , Schlichting I , Yumerefendi H , et al. LOVTRAP: an optogenetic system for photoinduced protein dissociation. Nat Methods. 2016;13:755–758. 27427858 10.1038/nmeth.3926 PMC5137947

[45] Strickland D , Yao X , Gawlak G , Rosen MK , Gardner KH , Sosnick TR . Rationally improving LOV domain‐based photoswitches. Nat Methods. 2010;7:623–626. 20562867 10.1038/nmeth.1473 PMC2914111

[46] Strickland D , Lin Y , Wagner E , Hope CM , Zayner J , Antoniou C , et al. TULIPs: tunable, light‐controlled interacting protein tags for cell biology. Nat Methods. 2012;9:379–384. 22388287 10.1038/nmeth.1904 PMC3444151

[47] Diensthuber RP , Engelhard C , Lemke N , Gleichmann T , Ohlendorf R , Bittl R , et al. Biophysical, mutational, and functional investigation of the chromophore‐binding pocket of light‐oxygen‐voltage photoreceptors. ACS Synth Biol. 2014;3:811–819. 24926890 10.1021/sb400205x

[48] Zayner JP , Sosnick TR . Factors that control the chemistry of the LOV domain photocycle. PLoS One. 2014;9:e87074. 24475227 10.1371/journal.pone.0087074 PMC3903614

[49] Takeuchi J , Chen H , Coffino P . Proteasome substrate degradation requires association plus extended peptide. EMBO J. 2007;26:123–131. 17170706 10.1038/sj.emboj.7601476 PMC1782366

[50] Sun W , Zhang W , Zhang C , Mao M , Zhao Y , Chen X , et al. Light‐induced protein degradation in human‐derived cells. Biochem Biophys Res Commun. 2017;487:241–246. 28412349 10.1016/j.bbrc.2017.04.041

[51] Leibowitz ML , Papathanasiou S , Doerfler PA , Blaine LJ , Sun L , Yao Y , et al. Chromothripsis as an on‐target consequence of CRISPR‐Cas9 genome editing. Nat Genet. 2021;53:895–905. 33846636 10.1038/s41588-021-00838-7 PMC8192433

[52] Chauhan VP , Sharp PA , Langer R . Altered DNA repair pathway engagement by engineered CRISPR‐Cas9 nucleases. Proc Natl Acad Sci USA. 2003;120:e2300605120. 10.1073/pnas.2300605120 PMC10242711 36881621

[53] Riesenberg S , Kanis P , Macak D , Wollny D , Düsterhöft D , Kowalewski J , et al. Efficient high‐precision homology‐directed repair‐dependent genome editing by HDRobust. Nat Methods. 2023;20:1388–1399. 37474806 10.1038/s41592-023-01949-1 PMC10482697

[54] Petrenčáková M , Varhač R , Kožár T , Nemergut M , Jancura D , Schwer MS , et al. Conformational properties of LOV2 domain and its C450A variant within broad pH region. Biophys Chem. 2020;259:106337. 32126442 10.1016/j.bpc.2020.106337

[55] Richter G , Weber S , Römisch W , Bacher A , Fischer M , Eisenreich W . Photochemically induced dynamic nuclear polarization in a C450A mutant of the LOV2 domain of the *Avena sativa* blue‐light receptor phototropin. J Am Chem Soc. 2005;127:17245–17252. 16332073 10.1021/ja053785n

[56] Bae S , Park J , Kim JS . Cas‐OFFinder: a fast and versatile algorithm that searches for potential off‐target sites of Cas9 RNA‐guided endonucleases. Bioinformatics. 2014;30:1473–1475. 24463181 10.1093/bioinformatics/btu048 PMC4016707

