# Supplementary material for: Engineered AcrIIA5 for optogenetic control of CRISPR‐Cas9‐based genome editing
Source: mLife. 2025 Dec 12;4(6):697–708. doi: 10.1002/mlf2.70016 (PMC12754625; doi:10.1002/mlf2.70016)
Supplement: Supplementary file 1 — Revised Supporting Information. [file MLF2-4-697-s001.pdf]

Supporting Information

Table of contents

Figures

Figure S1. Normalized indel formation efficiency for SpCas9 with AcrIIA5-AsLOV2 hybrids.

Figure S2. Analysis of blue light-mediated indel formation for SpCas9 with hybrid L109.

Figure S3. Schematic of the newly designed CN-A5 variants derivated from the hybrid L109.

Figure S4. Normalized indel formation efficiency for SpCas9 with newly designed CN-A5 variants.

Figure S5. Analysis of blue light-mediated indel formation for SpCas9 with CN-A5-n10.

Figure S6. Structure of AsLOV2 in the dark state.

Figure S7. Normalized indel formation efficiency for SpCas9 with mutated CN-A5.

Figure S8. Normalized indel formation efficiency for Cas9 orthologs with CN-A5 variants.

Figure S9. The off-targeting examination.

Figure S10. The comparison of the inhibition ability of AcrIIA5 and hybrid C-ter.

Figure S11. Blue light control of AcrIIA5-LOV9 and its mutated variants.

Tables

Table S1. Mutations introduced to AsLOV2.

Table S1. Target sgRNA-protospacer sequences in this study.

Table S3. Off target sgRNA-protospacer sequences in this study.

Sequences

Sequence S1. Plasmids (functional amino acid sequences shown only) used in this study.

Sequence S2. Plasmids (functional nucleotide sequences shown only) used in this study.

References

Figures

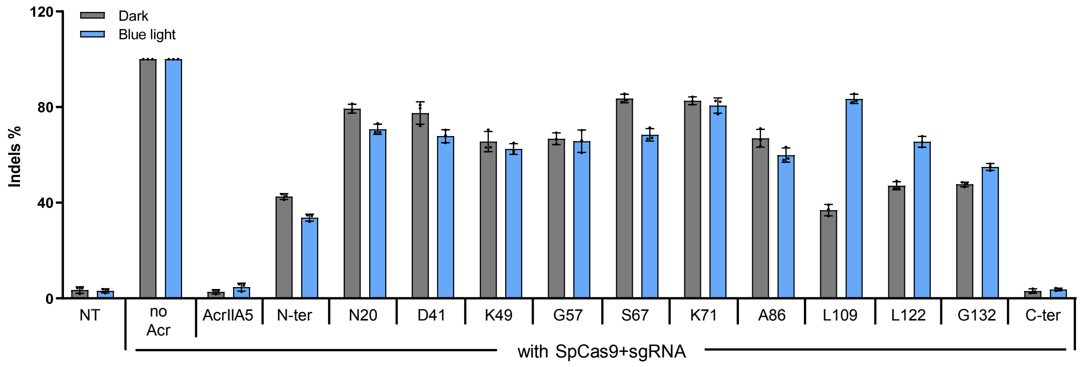

Figure S1. Normalized indel formation efficiency for SpCas9 with AcrIIA5-AsLOV2 hybrids. The plasmids expressing SpCas9, AcrIIA5-AsLOV2 hybrids, and the sgRNA targeting the CCR5 locus were co-transfected into HEK293T cells. The vector mole ratio of the AcrIIA5-AsLOV2 hybrids:SpCas9 is 1:1. At 12 h after transfection, the culture plate was placed below LED lamps for illumination, and the cells were cultured for an additional 48 h. Throughout the entire 60 h period, dark-treated cells were wrapped in tinfoil. NT means not transfected cells. The indel formation efficiency of SpCas9+sgRNA was normalized as 100%. All data are shown as individual data points and means ± s.d. for n = 3 biologically independent replicates.

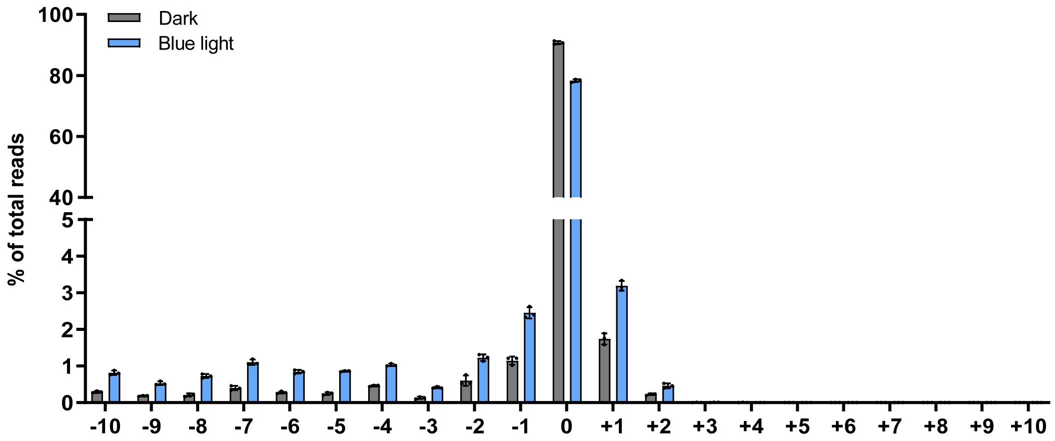

Figure S2. Analysis of blue light-mediated indel formation for SpCas9 with hybrid L109. The plasmids expressing SpCas9, hybrid L109, and the sgRNA targeting the CCR5 locus were co-transfected into HEK293T cells. The vector mole ratio of the hybrid L109:SpCas9 is 1:1. At 12 h after transfection, the culture plate was placed below LED lamps for illumination, and the cells were cultured for an additional 48 h. Throughout the entire 60 h period, dark-treated cells were wrapped in tinfoil. The data represent the percentage of aligned reads with an insertion or deletion of the given length. All data are shown as individual data points and means ± s.d. for n = 3 biologically independent replicates.

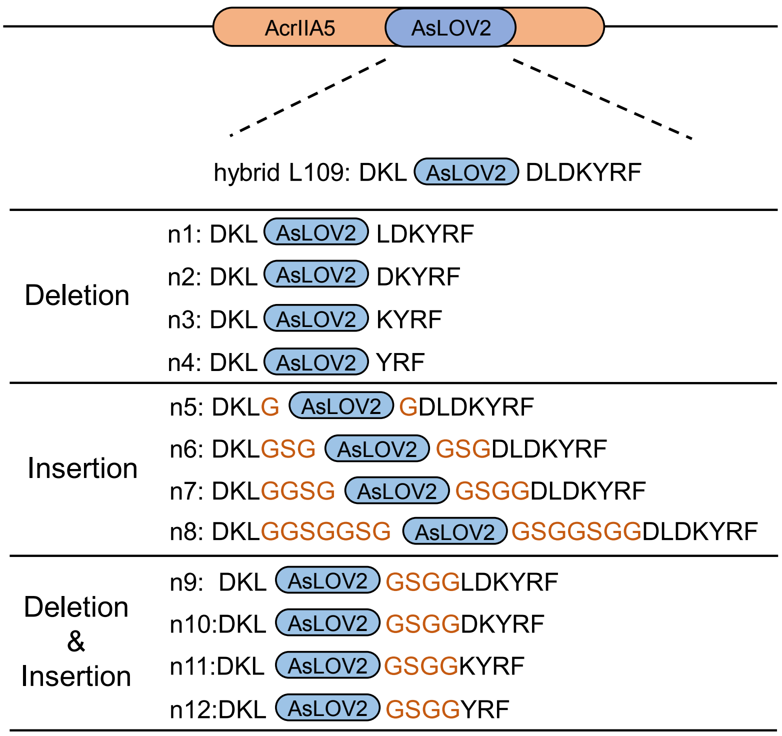

Figure S3. Schematic of the newly designed CN-A5 variants derivated from the hybrid L109. The amino acid sequences surrounding AsLOV2 are shown.

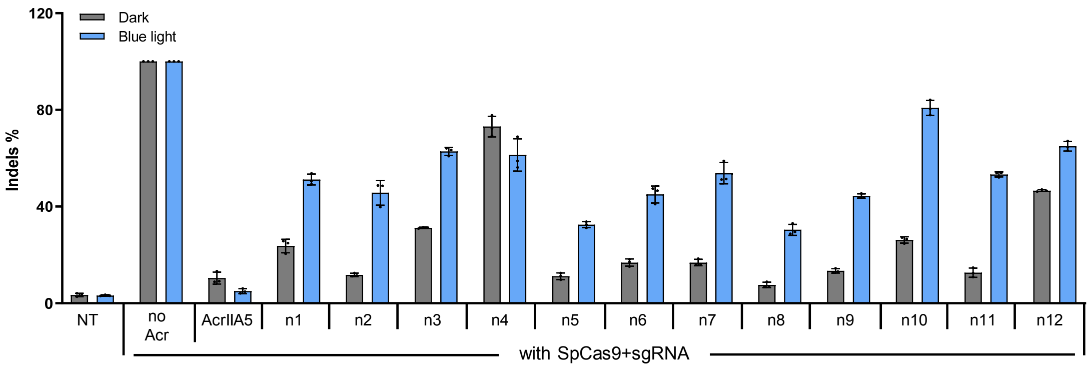

Figure S4. Normalized indel formation efficiency for SpCas9 with newly designed CN-A5 variants. The plasmids expressing SpCas9, the newly designed CN-A5 variants, and the sgRNA targeting the CCR5 locus were co-transfected into HEK293T cells. The vector mole ratio of the CN-A5 variants:SpCas9 is 1:1. At 12 h after transfection, the culture plate was placed below LED lamps for illumination, and the cells were cultured for an additional 48 h. Throughout the entire 60 h period, dark-treated cells were wrapped in tinfoil. NT means not transfected cells. The indel formation efficiency of SpCas9+sgRNA was normalized as 100%. All data are shown as individual data points and means ± s.d. for $n = 3$ biologically independent replicates.

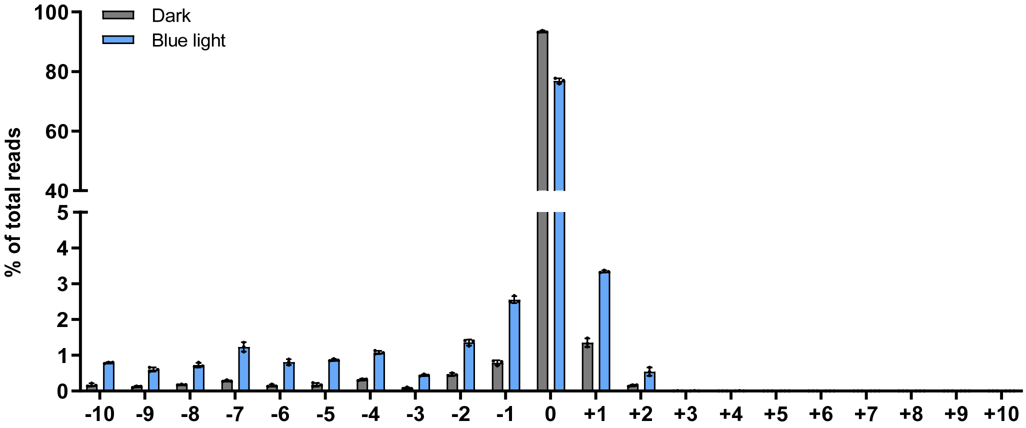

Figure S5. Analysis of blue light-mediated indel formation for SpCas9 with CN-A5-n10. The plasmids expressing SpCas9, CN-A5-n10, and the sgRNA targeting the CCR5 locus were co-transfected into HEK293T cells. The vector mole ratio of the CN-A5-n10:SpCas9 is 1:1. At 12 h after transfection, the culture plate was placed below LED lamps for illumination, and the cells were cultured for an additional 48 h. Throughout the entire 60 h period, dark-treated cells were wrapped in tinfoil. The data represent the percentage of aligned reads with an insertion or deletion of the given length. All data are shown as individual data points and means ± s.d. for n = 3 biologically independent replicates.

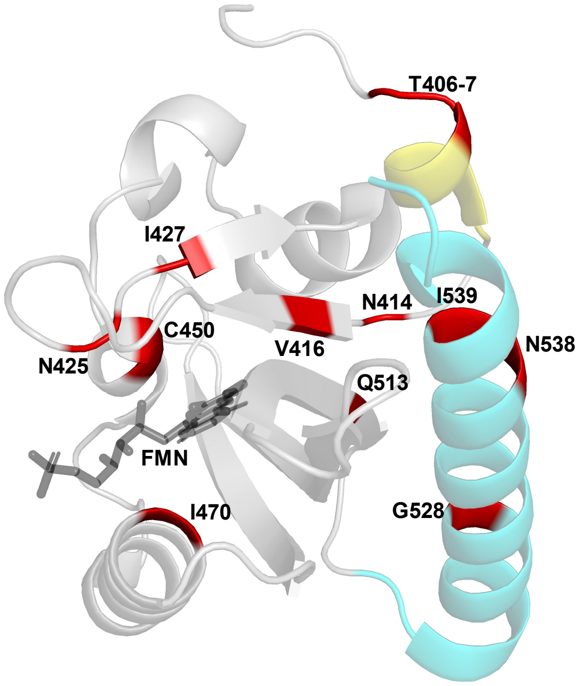

Figure S6. Structure of AsLOV2 in the dark state. The AsLOV2 consists of PAS core (light gray), FMN (dark gray), N-terminal A'α helix (yellow), and C-terminal Jα helix (blue). (PDB code 7PGX). The residues to be mutated are marked in red.

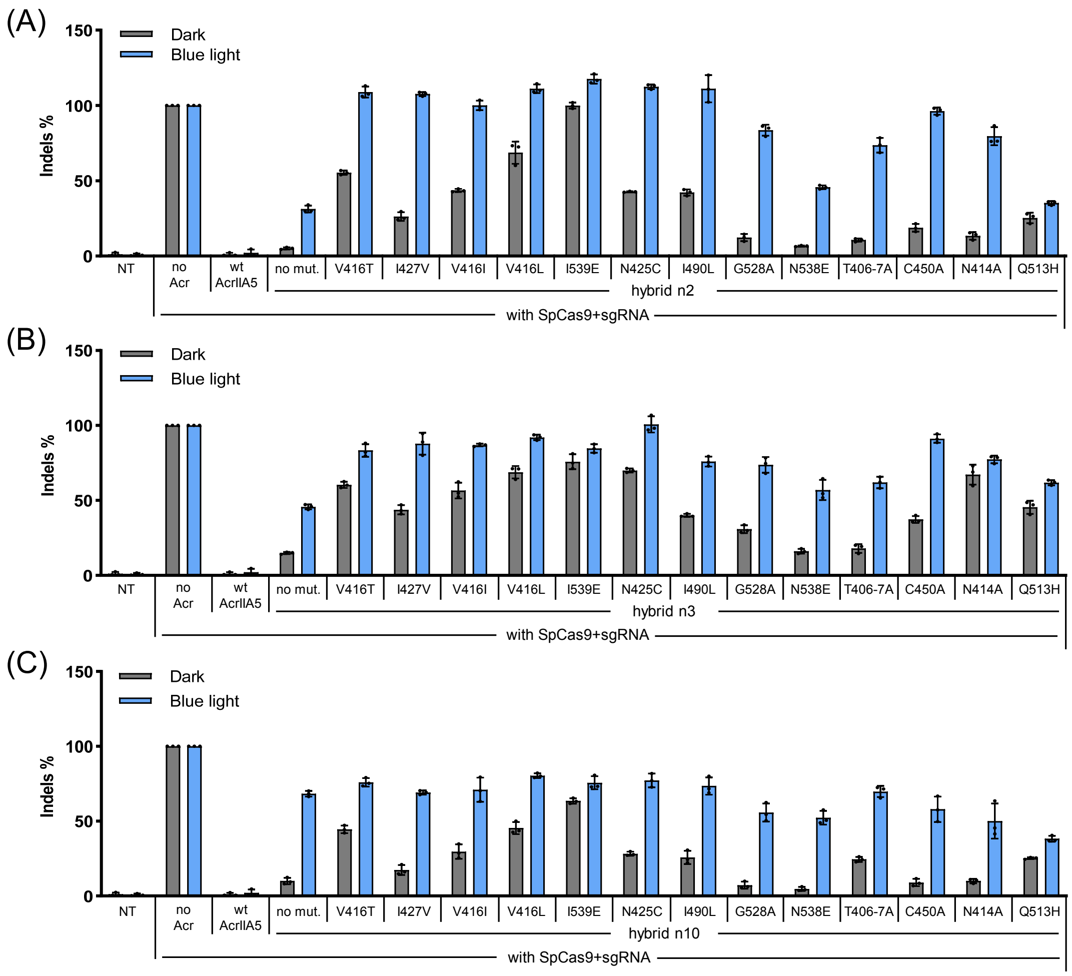

Figure S7. Normalized indel formation efficiency for SpCas9 with mutated CN-A5. (A–C) Blue light control of (A) mutated CN-A5-n2, (B) mutated CN-A5-n3, and (C) mutated CN-A5-n10. The plasmids expressing SpCas9, CN-A5 variants, and the sgRNA targeting the CCR5 locus were co-transfected into HEK293T cells. The vector mole ratio of the CN-A5 variants:SpCas9 is 1:1. At 12 h after transfection, the culture plate was placed below LED lamps for illumination, and the cells were cultured for an additional 48 h. Throughout the entire 60 h period, dark-treated cells were wrapped in tinfoil. The indel formation efficiency of SpCas9+sgRNA was normalized as 100%. NT means not transfected cells. No mut. menas no mutation. All data are shown as individual data points and means ± s.d. for $n = 3$ biologically independent replicates.

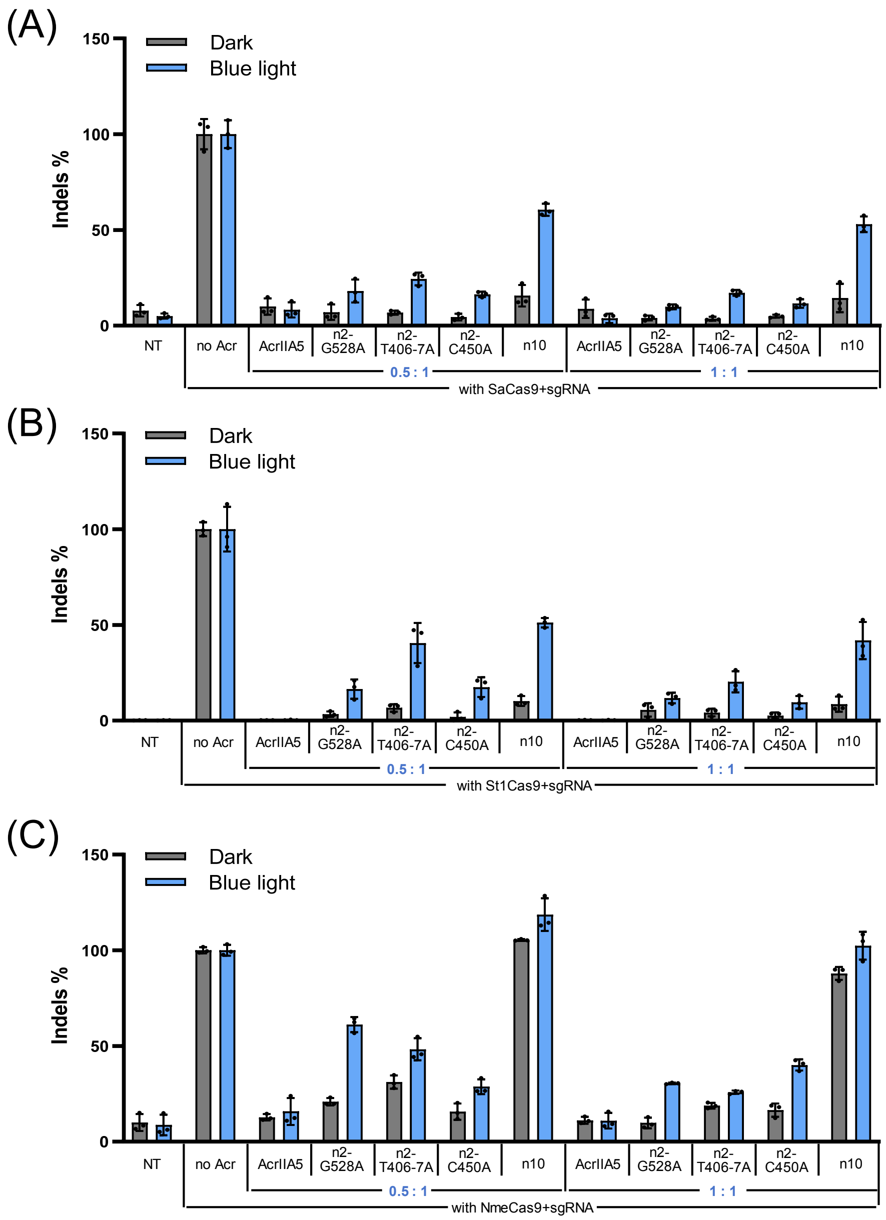

Figure S8. Normalized indel formation efficiency for Cas9 orthologs with CN-A5 variants. (A–C) Blue light control of CN-A5 variants n2-G528A, n2-T406-7A, n2-C450A and n10 to (A) SaCas9, (B) St1Cas9, and (C) NmeCas9. The plasmids expressing Cas9 orthologs, CN-A5 variants, and sgRNA were co-transfected into HEK293T cells. The vector mole ratio of the CN-A5 variants:Cas9 orthologs is indicated in blue-marked numbers below the x-coordinate. At 12 h after transfection, the culture plate was placed below LED lamps for illumination, and the cells were cultured for an additional 48 h. Throughout the entire 60 h period, dark-treated cells were wrapped in tinfoil. NT means not transfected. The indel formation efficiency of Cas9+sgRNA was normalized as 100%. All data are shown as individual data points and means ± s.d. for $n = 3$ biologically independent replicates.

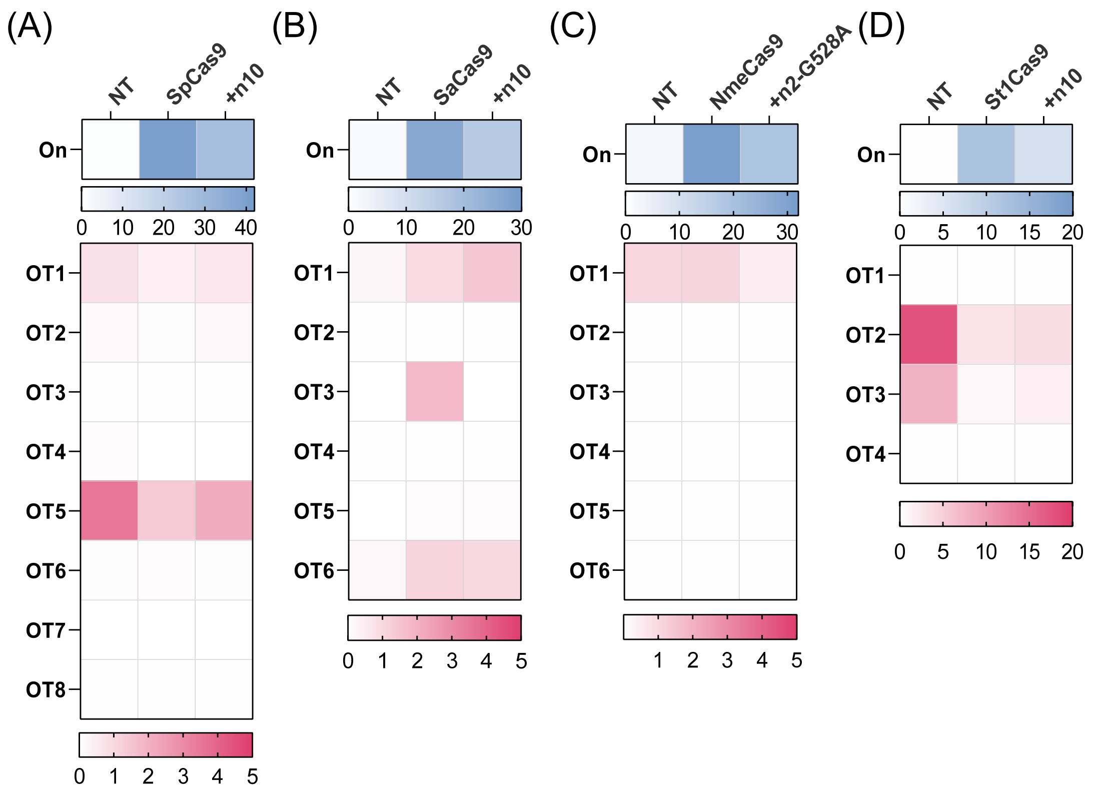

Figure S9. The off-targeting examination. Possible off-target loci were predicted by Cas-Offinder. The blue heat maps in the above represent the on-target indel formation efficiency and the red heat maps represent the off-target editing. (A) HEK293T cells expressing SpCas9, CN-A5-n10, and the sgRNA targeting the CCR5 locus were irradiated with blue light. The vector mole ratio of the CN-A5-n10:SpCas9 is 1:1. (B) HEK293T cells expressing SaCas9, CN-A5-n10, and the sgRNA targeting the Sa-site6 locus were irradiated with blue light. The vector mole ratio of the CN-A5-n10:SaCas9 is 0.5:1. (C) HEK293T cells expressing NmeCas9, CN-A5-n2-G528A, and the sgRNA targeting the DYRK1A locus were irradiated with blue light. The vector mole ratio of the CN-A5-n2-G528A:NmeCas9 is 0.5:1. (D) HEK293T cells expressing St1Cas9, CN-A5-n10, and the sgRNA targeting the DYRK1A locus were irradiated with blue light. The vector mole ratio of the CN-A5-n10:NmeCas9 is 0.5. NT means not transfected. All data represent the average of efficiency for $n = 3$ biologically independent replicates.

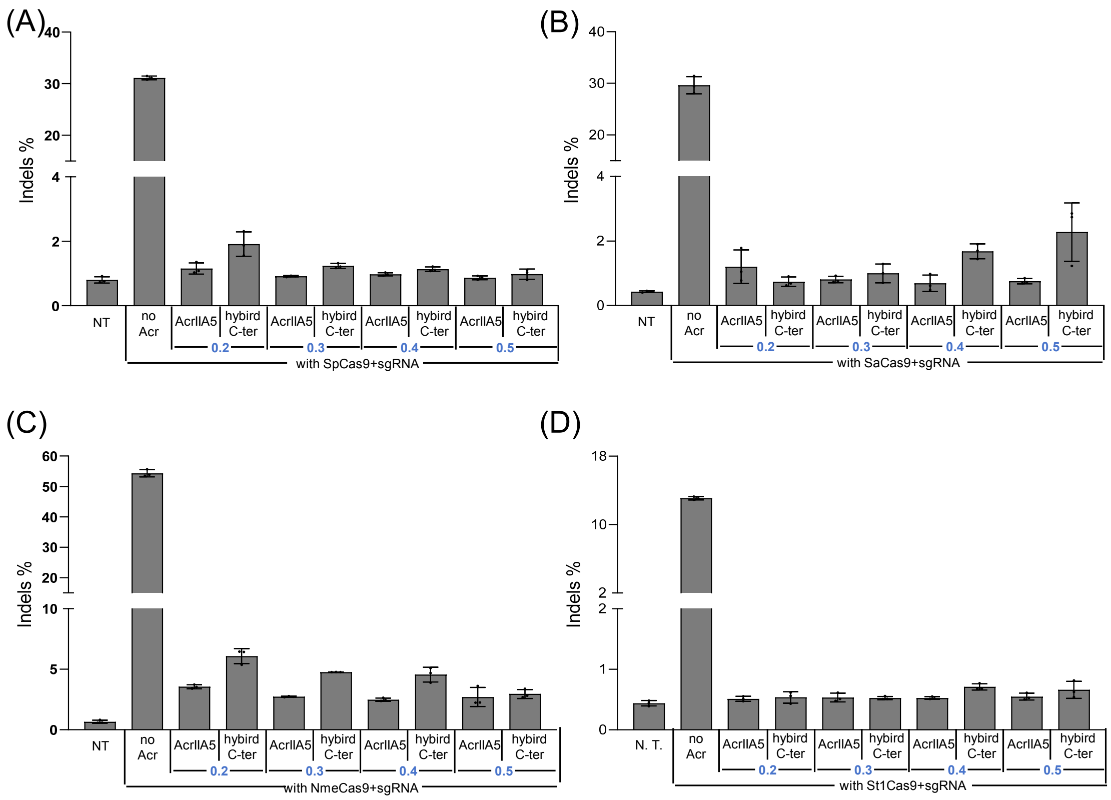

Figure S10. The comparison of the inhibition ability of AcrIIA5 and hybrid C-ter. (A–D) SpCas9 (A), SaCas9 (B), NmeCas9 (C), and St1Cas9 (D) were tested. The plasmids expressing Cas9 orthologs, AcrIIA5-AsLOV2 hybrids, and sgRNA were co-transfected into HEK293T cells. The mole ratio of AcrIIA5-AsLOV2 hybrids:Cas9 were indicated in blue number. The transfected cells were wrapped in tinfoil and cultured for 60 h. NT means not transfected cells. All data are shown as individual data points and means ± s.d. for n = 3 biologically independent replicates.

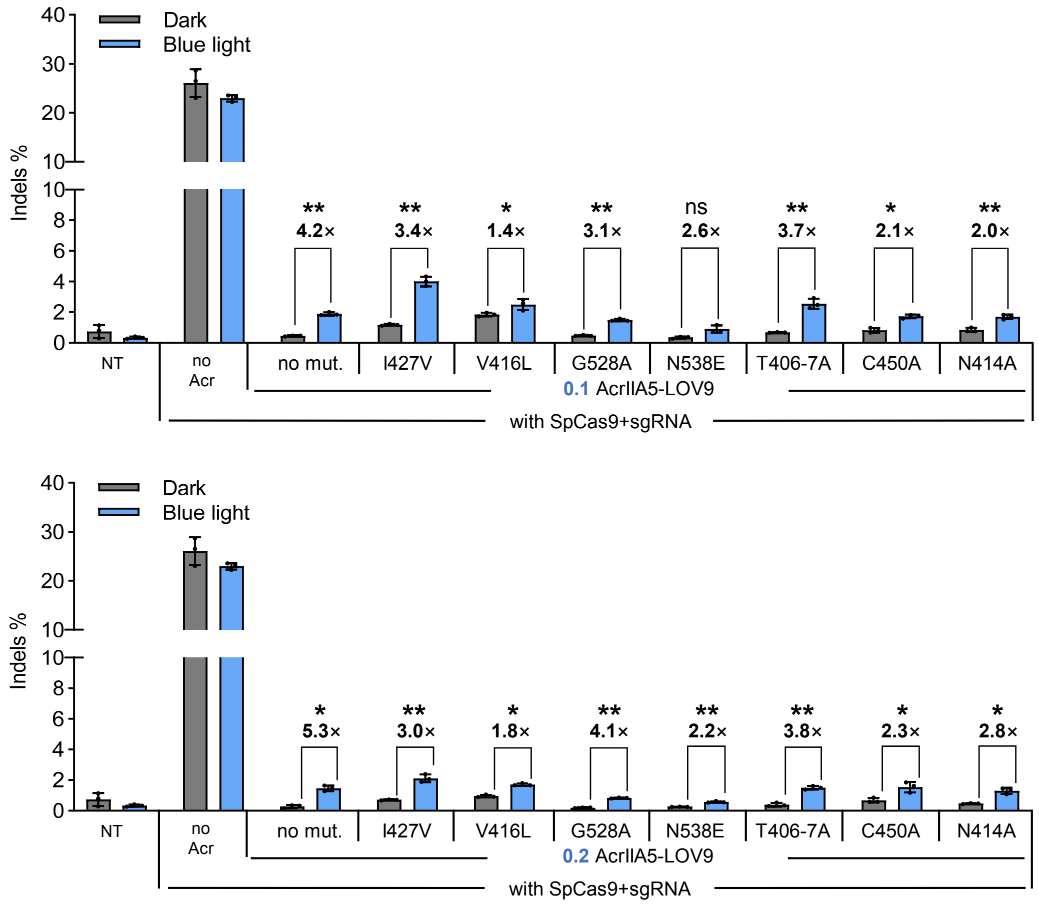

Figure S11. Blue light control of AcrIIA5-LOV9 and its mutated variants. The plasmids expressing SpCas9, AcrIIA5-LOV9 variants, and the sgRNA targeting the CCR5 locus were co-transfected into HEK293T cells. The mole ratio of AcrIIA5-LOV9:Cas9 is indicated in blue number. At 12 h after transfection, the culture plate was placed below LED lamps for illumination, and the cells were cultured for an additional 48 h. Throughout the entire 60 h period, dark-treated cells were wrapped in tinfoil. All data are shown as individual data points and means ± s.d. for $n = 3$ biologically independent replicates. P values (*$P < 0.05$; **$P < 0.01$; ***$P < 0.001$; ns means not significant) are calculated using the two-tailed Student's t-test.

Tables

Table S1. Mutations introduced to AsLOV2

Affect	Mutation	Description	Reference
Photocycle	V416T	Shorten the photocycle	1
	I427V	Shorten the photocycle	
	V416I	Increase the photocycle	
	V416L	Increase the photocycle	
	N425C	Shorten the photocycle	2
	I470L	Shorten the photocycle	
	N414A	Increase the photocycle	3
	Q513H	Shorten the photocycle	
	C450A	Maintain AsLOV2 in the dark state	4
	I539E	Maintain AsLOV2 in the lit state	5
Affinity of A'α or Jα to the PAS core	G528A	Stabilize the Jα interaction	6
	N538E	Stabilize the Jα interaction	
	T406-7A	Stabilize the N-terminal A'α helix of AsLOV2 and enhance Jα docking affinity	7

Table S2. Target sgRNA-protospacer sequences in this study.

locus	Target sgRNA-protospacer sequence	PAM	Oligo primer for HTS (5'→3')	
CCR5	gTGACATCAATTATTATACAT	TGG (SpCas9)	F-primer	ATGCACAGGGTGGAACAAGATGG
			R-primer	TAGATGTCAGTCATGCTCTTCAGCC
Sa-site6	GATGTTCCAATCAGTACGCA	GAGAGT (SaCas9)	F-primer	GCGGGGTCCCAGGTGCTGAC
			R-primer	TAGCATTGCAGAGAGGCGTATC
DYRK1A	gTTTAATGGTATAGAAGATCCA	TAGTGAT (NmeCas9)	F-primer	AGGTTGTTGCTGTTGCTTTAC
			R-primer	GTATCATTTGACATATCTAATGGTTCC
DYRK1A	gTGAATCTGGTCAGAATATGATAAG	GCAGAA (St1Cas9)	F-primer	TACAGTGACCGTCGCCAGCC
			R-primer	TCCCAATCCATAATCCCACGTTGC

Table S3. Off target sgRNA-protospacer sequences in this study.

Locus	Off target sgRNA-protospacer sequence	PAM	Oligo primers for HTS (5'→3')	
SpCas9 -OT1	TGAaATCAATTtTTATACtT	GGG	F-primer	ACCTCTATCTCGAAGCTTGGA
			R-primer	CATAGGTATGACTTTGATTCTAGC
SpCas9 -OT2	TGAtATaAATTATTATAtAT	GGG	F-primer	GAATTGTAGCTAGGATCCATGC
			R-primer	CACTTGTTTACATCTGTAAAGACC
SpCas9 -OT3	TGACATaAATTATgAaACAT	TGG	F-primer	GTATGTCCAGCTCTAGCCGC
			R-primer	CCACTGACAAGGTCTTCTTCC
SpCas9 -OT4	TGACATaAATTATTATAagT	AGG	F-primer	GTAACCACCTTGAATGTGATGT
			R-primer	GATACCTACCAGAGTAAGCATC
SpCas9 -OT5	TGACATaAATTATTtTACtT	AGG	F-primer	ATCGGTCAGGGTAGTGCAGG
			R-primer	GTATTTGGAAGAATGGTGACTTG
SpCas9 -OT6	TtACATCAATTtTTgTACAT	TGG	F-primer	CATATGGTATTAGAGAATAACAAG
			R-primer	TACAAAGTAGGGTGGATTCAAG
SpCas9 -OT7	TGACcTCAtTTATTAaACAT	AGG	F-primer	GTCACTGTTATAAAAGGGATTGA
			R-primer	GATAATCAGCAGATATTTTAACCC
SpCas9 -OT8	TGAaATgAATTATTAaACAT	TGG	F-primer	TGAATATATACAAAGCTTCTGAAC
			R-primer	CAAGGCCCAATCGGATACAAGATA
SaCas9 -OT1	aATGTcCCAATCAGaAgGCA	CAGAGT	F-primer	CTGAATAATACAGACCAGTGACC
			R-primer	GTCATTGCTTGTGAGATCAGTC
SaCas9 -OT2	GAgtTTaCAATCtGTACGCA	CAGAGT	F-primer	CACCTGCTGGCATCGTGGAG
			R-primer	ACACACCGTGACGGGGTCCT
SaCas9 -OT3	GAgGcTCCAATaAGTACtCA	ATGAAT	F-primer	CCATAGGCCAAGCCCATCCA
			R-primer	CCAACCTGGATCAAGTGGAAG
SaCas9 -OT4	GATaTTggAATCAGTAaGCA	AAGAAT	F-primer	TGTCCAGTTCTTAACAAAAAGTCA
			R-primer	GTCTTTATCCCTTCATTGATAGTC
SaCas9 -OT5	GATGTgCtAATCAGTACcCt	GCGAAT	F-primer	TGACAACAGGCACTATTCCC
			R-primer	CCCCAGCTTGTTGCTTTGAG
SaCas9 -OT6	GATtTTCtcATCAGTAaGCA	TGGAAT	F-primer	AAAGACAGCCTAAACCATTCTC
			R-primer	CTGGACTCAAGTGATCTACCCA

NmeCas9-OT1	aTTgATGGTATAaAAGATCCcA	AACGATT	F-primer	GCCCTGGTTCTAGGGAAGAG
			R-primer	TGTCAGACAATGTAGGAGCC
NmeCas9-OT2	TTTAATaccATAGAAtATCCAA	ATTGATT	F-primer	TGGGATTTGGGGGTATATGTTT
			R-primer	TTTTGATCGATTTCAGAAACATG
NmeCas9-OT3	cTTAATGGTATAGAAGtcaCA	GGAGGATT	F-primer	CCAGCGAGTTCTGGGCAGGT
			R-primer	AGGCTGGAGATAGAACCTGT
NmeCas9-OT4	TTgtATtGTATAGAAcATCCA	TCCTGATT	F-primer	GTGCCACCCTCACACTTGCCA
			R-primer	CCTTGAAGGTGATGGGTATGTC
NmeCas9-OT5	TgTAATGGTATtcAAGcTCCA	GGAAGATT	F-primer	GCAGCCGCAAAGCATGAAGT
			R-primer	CACCTAAGAGGTAGGTGAGGCG
NmeCas9-OT6	TTTtATGGTcTAGAAttTCCA	AATTGATT	F-primer	AGTCCCACAGAGGGGTCCAG
			R-primer	TTCTTCCTGCTTAACCCTCTTC
St1Cas9-OT1	TGAgTCTGGTCAGcATATGAaAAt	ATAGAAA	F-primer	ACTATGTTGTTGTCTGCCCC
			R-primer	CAGGGGTGTATCATAGAAGAGA
St1Cas9-OT2	TGAcTCcaGaCtGAATATGATAAG	TGAGAAA	F-primer	ATCAGGGAAATGTCACACTCT
			R-primer	GGTCTTAGTGCTGTATCCACTT
St1Cas9-OT3	TcAATaTGGTgAtAATATGccAAG	GGAGAAA	F-primer	TAACATGTTGATGGTATTCAGTGA
			R-primer	TGTTTGGGGGGCCACAAACC
St1Cas9-OT4	atAATCTGcTCAGAATtTatTAAG	AAAGAAT	F-primer	AGATGCCCTGCCCAGAAAGC
			R-primer	TCAATATAGTTTGTCACGCAGG

Sequences

Sequence S1.

Plasmids (functional amino acid sequences shown only) used in this study.

pCMV-SpCas9 (SpCas9-NLS)

MDKKYSIGLAIGTNSVGWAVITDEYKVPSKKFKVLGNTDRHSIKKNLIGALLFDSGETAEATRLKRTAR
RRYTRRKNRICYLQEIFSNEMAKVDDSFFHRLEESFLVEEDKKHERHPIFGNIVDEVAYHEKYPTIYHL
RKKLVDSTDKADLRLIYLALAHMIKFRGHFLIEGDLNPDNSDVDKLFIQLVQTYNQLFEENPINASGVD
AKAILSARLSKSRRLENLIAQLPGEKKNGLFGNLIALSLGLTPNFKSNFDLAEDAKLQLSKDTYDDDLD
NLLAQIGDQYADLFLAAKNLSDAILLSDILRVNTEITKAPLSASMIKRYDEHHQDLTLLKALVRQQLPE
KYKEIFFDQSKNGYAGYIDGGASQEEFYKFIKPILEKMDGTEELLVKLNREDLLRKQRTFDNGSIPHQI
HLGELHAILRRQEDFYPFLKDNREKIEKILTFRIPYYVGPLARGNSRFAWMTRKSEETITPWNFEEVVD
KGASAQSFIERMTNFDKNLPNEKVLPKHSLLYEYFTVYNELTKVKYVTEGMRKPAFLSGEQKKAIVDLL
FKTNRKVTVKQLKEDYFKKIECFDSVEISGVEDRFNASLGTYHDLLKIIKDKDFLDNEENEDILEDIVL
TLTLFEDREMIEERLKTYAHLFDDKVMKQLKRRRYTGWGRLSRKLINGIRDKQSGKTILDFLKSDGFAN
RNFMQLIHDDSLTFKEDIQKAQVSGQGDSLHEHIANLAGSPAIKKGILQTVKVVDELVKVMGRHKPENI
VIEMARENQTTQKGQKNSRERMKRIEEGIKELGSQILKEHPVENTQLQNEKLYLYYLQNGRDMYVDQEL
DINRLSDYDVDHIVPQSFLKDDSIDNKVLTRSDKNRGKSDNVPSEEVVKKMKNYWRQLLNAKLITQRKF
DNLTKAERGGLSELDKAGFIKRQLVETRQITKHVAQILDSRMNTKYDENDKLIREVKVITLKSKLVSDF
RKDFQFYKVREINNYHHAHDAYLNAVVGTALIKKYPKLESEFVYGDYKVYDVRKMIAKSEQEIGKATAK
YFFYSNIMNFFKTEITLANGEIRKRPLIETNGETGEIVWDKGRDFATVRKVLSMPQVNIVKKTEVQTGG
FSKESILPKRNSDKLIARKKDWDPKKYGGFDSPTVAYSVLVVAKVEKGKSKKLKSVKELLGITIMERSS
FEKNPIDFLEAKGYKEVKKDLIIKLPKYSLFELENGRKRMLASAGELQKGNELALPSKYVNFLYLASHY
EKLKGSPEDNEQKQLFVEQHKHYLDEIIEQISEFSKRVILADANLDKVLSAYNKHRDKPIREQAENIIH
LFTLTNLGAPAAFKYFDTTIDRKRYTSTKEVLDATLIHQSITGLYETRIDLSQLGGDSGGSPKKKRKV*

pCMV-SaCas9 (SaCas9-NLS)

MGKRNYILGLAIGITSVGYGIIDYETRDVIDAGVRLFKEANVENNEGRRSKRGARRLKRRRRHRIQRVK
KLLFDYNLLTDHSELSGINPYEARVKGLSQKLSEEEFSAALLHLAKRRGVHNVNEVEEDTGNELSTKEQ
ISRNSKALEEKYVAELQLERLKKDGEVRGSINRFKTSDYVKEAKQLLKVQKAYHQLDQSFIDTYIDLLE
TRRTYYEGPGEGSPFGWKDIKEWYEMLMGHCTYFPEELRSVKYAYNADLYNALNDLNNLVITRDENEKL
EYYEKFQIIENVFKQKKKPTLKQIAKEILVNEEDIKGYRVTSTGKPEFTNLKVYHDIKDITARKEIIEN
AELLDQIAKILTIYQSSEDIQEELTNLNSELTQEEIEQISNLKGYTGTHNLSLKAINLILDELWHTNDN
QIAIFNRLKLVPKKVDLSQQKEIPTTLVDDFILSPVVKRSFIQSIKVINAIIKKYGLPNDIIIELAREK
NSKDAQKMINEMQKRNRQTNERIEEIIRTTGKENAKYLIEKIKLHDMQEGKCLYSLEAIPLEDLLNNPF
NYEVDHIIPRSVSFDNSFNNKVLVKQEENSKKGNRTPFQYLSSSDSKISYETFKKHILNLAKGKGRISK
TKKEYLLEERDINRFSVQKDFINRNLVDTRYATRGLMNLLRSYFRVNNLDVKVKSINGGFTSFLRRKWK
FKKERNKGYKHHAEDALIIANADFIFKEWKKLDKAKKVMENQMFEEKQAESMPEIETEQEYKEIFITPH
QIKHIKDFKDYKYSHRVDKKPNRELINDTLYSTRKDDKGNTLIVNNLNGLYDKDNDKLKKLINKSPEKL
LMYHHDPQTYQKLKLIMEQYGDEKNPLYKYYEETGNYLTKYSKKDNGPVIKKIKYYGNKLNAHLDITDD
YPNSRNKVVKLSLKPYRFDVYLDNGVYKFVTVKNLDVIKKENYYEVNSKCYEEAKKLKKISNQAEFIAS
FYNNDLIKINGELYRVIGVNNDLLNRIEVNMIDITYREYLENMNDKRPPRIIKTIASKTQSIKKYSTDI
LGNLYEVKSKKHPQIIKKGSGGSPKKKRKV*

pCMV-NmeCas9 (NmeCas9-NLS)

MAAFKPNSINYILGLAIGIASVGWAMVEIDEEENPIRLIDLGVRVFERAEVPKTGDSLAMARRLARSVR
RLTRRRAHRLLRTRRLLKREGVLQAANFDENGLIKSLPNTPWQLRAAALDRKLTPLEWSAVLLHLIKHR
GYLSQRKNEGETADKELGALLKGVAGNAHALQTGDFRTPAELALNKFEKESGHIRNQRSDYSHTFSRKD
LQAELILLFEKQKEFGNPHVSGGLKEGIETLLMTQRPALSGDAVQKMLGHCTFEPAEPKAAKNTYTAER
FIWLTKLNNLRILEQGSERPLTDTERATLMDEPYRKSKLTYAQARKLLGLEDTAFFKGLRYGKDNAEAS
TLMEMKAYHAISRALEKEGLKDKKSPLNLSPELQDEIGTAFSLFKTDEDITGRLKDRIQPEILEALLKH
ISFDKFVQISLKALRRIVPLMEQGKRYDEACAEIYGDHYGKKNTEEKIYLPPIPADEIRNPVVLRALSQ
ARKVINGVVRRYGSPARIHIETAREVGKSFKDRKEIEKRQEENRKDREKAAAKFREYFPNFVGEPKSKD
ILKLRLYEQQHGKCLYSGKEINLGRLNEKGYVEIDHALPFSRTWDDSFNNKVLVLGSENQNKGNQTPYE
YFNGKDNSREWQEFKARVETSRFPRSKKQRILLQKFDEDGFKERNLNDTRYVNRFLCQFVADRMRLTGK
GKKRVFASNGQITNLLRGFWGLRKVRAENDRHHALDAVVVACSTVAMQQKITRFVRYKEMNAFDGKTID
KETGEVLHQKTHFPQPWEFFAQEVMIRVFGKPDGKPEFEEADTLEKLRTLLAEKLSSRPEAVHEYVTPL
FVSRAPNRKMSGQGHMETVKSAKRLDEGVSVLRVPLTQLKLKDLEKMVNREREPKLYEALKARLEAHKD
DPAKAFAEPFYKYDKAGNRTQQVKAVRVEQVQKTGVWVRNHNGIADNATMVRVDVFEKGDKYYLVPIYS
WQVAKGILPDRAVVQGKDEEDWQLIDDSFNFKFSLHPNDLVEVITKKARMFGYFASCHRGTGNINIRIH
DLDHKIGKNGILEGIGVKTALSFQKYQIDELGKEIRPCRLKKRPPVRSGGSPKKKRKV*

pCMV-St1Cas9 (St1Cas9-NLS)

MGSDLVLGLAIGIGSVGVGILNKVTGEIIHKNSRIFPAAQAENNLVRRTNRQGRRLARRKKHRRVRLNR
LFEESGLITDFTKISINLNPYQLRVKGLTDELSNEELFIALKNMVKHRGISYLDDASDDGNSSVGDYAQ
IVKENSKQLETKTPGQIQLERYQTYGQLRGDFTVEKDGKKHRLINVFPTSAYRSEALRILQTQQEFNPQ
ITDEFINRYLEILTGKRKYYHGPGNEKSRTDYGRYRTSGETLDNIFGILIGKCTFYPDEFRAAKASYTA
QEFNLLNDLNNLTVPTETKKLSKEQKNQIINYVKNEKAMGPAKLFKYIAKLLSCDVADIKGYRIDKSGK
AEIHTFEAYRKMKTLETLDIEQMDRETLDKLAYVLTLNTEREGIQEALEHEFADGSFSQKQVDELVQFR
KANSSIFGKGWHNFSVKLMMELIPELYETSEEQMTILTRLGKQKTTSSSNKTKYIDEKLLTEEIYNPVV
AKSVRQAIKIVNAAIKEYGDFDNIVIEMARETNEDDEKKAIQKIQKANKDEKDAAMLKAANQYNGKAEL
PHSVFHGHKQLATKIRLWHQQGERCLYTGKTISIHDLINNSNQFEVDHILPLSITFDDSLANKVLVYAT
ANQEKGQRTPYQALDSMDDAWSFRELKAFVRESKTLSNKKKEYLLTEEDISKFDVRKKFIERNLVDTRY
ASRVVLNALQEHFRAHKIDTKVSVVRGQFTSQLRRHWGIEKTRDTYHHHAVDALIIAASSQLNLWKKQK
NTLVSYSEDQLLDIETGELISDDEYKESVFKAPYQHFVDTLKSKEFEDSILFSYQVDSKFNRKISDATI
YATRQAKVGKDKADETYVLGKIKDIYTQDGYDAFMKIYKKDKSKFLMYRHDPQTFEKVIEPILENYPNK
QINEKGKEVPCNPFLKYKEEHGYIRKYSKKGNGPEIKSLKYYDSKLGNHIDITPKDSNNKVVLQSVSPW
RADVYFNKTTGKYEILGLKYADLQFEKGTGTYKISQEKYNDIKKKEGVDSDSEFKFTLYKNDLLLVKDT
ETKEQQLFRFLSRTMPKQKHYVELKPYDKQKFEGGEALIKVLGNVANSGQCKKGLGKSNISIYKVRTDV
LGNQHIIKNEGDKPKLDFSGGSPKKKRKV*

pCMV-AcrIIA5 (AcrIIA5-NLS)

MAYGKSRYNSYRKRSFNRSNKQRREYAQEMDRLEKAFENLDGWYLSSMKDSAYKDFGKYEIRLSNHSAD
NKYHDLENGRLIVNIKASKLNFVDIIENKLDKIIEKIDKLDLDKYRFINATNLEHDIKCYYKGFKTKKE
VISRADPKKKRKV*

pCMV-CASANOVA-A5 (N-AcrIIA5-AsLOV2-C-AcrIIA5-NLS)

MAYGKSRYNSYRKRSFNRSNKQRREYAQEMDRLEKAFENLDGWYLSSMKDSAYKDFGKYEIRLSNHSAD
NKYHDLENGRLIVNIKASKLNFVDIIENKLDKIIEKIDKLLATTLERIEKNFVITDPRLPDNPIIFASD
SFLQLTEYSREEILGRNCRFLQGPETDRATVRKIRDAIDNQTEVTVQLINYTKSGKKFWNLFHLQPMRD
QKGDVQYFIGVQLDGTEHVRDAAEREGVMLIKKTAENIDEAAKELDLDKYRFINATNLEHDIKCYYKGF
KTKKEVISRADPKKKRKV*

pCMV-CASANOVA-A5-n10 (N-AcrIIA5-AsLOV2-GSGG-C-AcrIIA5-NLS)

MAYGKSRYNSYRKRSFNRSNKQRREYAQEMDRLEKAFENLDGWYLSSMKDSAYKDFGKYEIRLSNHSAD
NKYHDLENGRLIVNIKASKLNFVDIIENKLDKIIEKIDKLLATTLERIEKNFVITDPRLPDNPIIFASD
SFLQLTEYSREEILGRNCRFLQGPETDRATVRKIRDAIDNQTEVTVQLINYTKSGKKFWNLFHLQPMRD
QKGDVQYFIGVQLDGTEHVRDAAEREGVMLIKKTAENIDEAAKELGSGGDKYRFINATNLEHDIKCYYK
GFKTKKEVISRADPKKKRKV*

pCMV-CASANOVA-A5-LOV9 (AcrIIA5-NLS-AsLOV2-cODC)

MAYGKSRYNSYRKRSFNRSNKQRREYAQEMDRLEKAFENLDGWYLSSMKDSAYKDFGKYEIRLSNHSAD
NKYHDLENGRLIVNIKASKLNFVDIIENKLDKIIEKIDKLDLDKYRFINATNLEHDIKCYYKGFKTKKE
VISRADPKKKRKVGGSLATTLERIEKNFVITDPRLPDNPIIFASDSFLQLTEYSREEILGRNCRFLQGP
ETDRATVRKIRDAIDNQTEVTVQLINYTKSGKKFWNLFHLQPMRDQKGDVQYFIGVQLDGTEHVRDAAE
REGVMLIKKTAENIDEAAASTLPVSCAWESGMKRHRAACASASINV*

Sequence S2.

Plasmids (functional nucleotide sequences shown only) used in this study.

pU6-SpCas9 sgRNA (U6 promoter-protospacer-SpCas9 sgRNA scaffold-polyT)

CACATTTCCCCGAAAAGTGCCACCTGACGTCGCTAGCTGTACAAAAAAGCAGGCTTTAAAGGAACCAAT
TCAGTCGACTGGATCCGGTACCAAGGTCGGGCAGGAAGAGGGCCTATTTCCCATGATTCCTTCATATTT
GCATATACGATACAAGGCTGTTAGAGAGATAATTAGAATTAATTTGACTGTAAACACAAAGATATTAGT
ACAAAATACGTGACGTAGAAAGTAATAATTTCTTGGGTAGTTTGCAGTTTTAAAATTATGTTTTAAAAT
GGACTATCATATGCTTACCGTAACTTGAAAGTATTTCGATTTCTTGGCTTTATATATCTTGTGGAAAGG
ACGAAACACCGTGACATCAATTATTATACATGTTTTAGAGCTAGAAATAGCAAGTTAAAATAAGGCTAG
TCCGTTATCAACTTGAAAAAGTGGCACCGAGTCGGTGCTTTTTTT

pU6-SaCas9 sgRNA (U6 promoter-protospacer-SaCas9 sgRNA scaffold-polyT)

CACATTTCCCCGAAAAGTGCCACCTGACGTCGCTAGCTGTACAAAAAAGCAGGCTTTAAAGGAACCAAT
TCAGTCGACTGGATCCGGTACCAAGGTCGGGCAGGAAGAGGGCCTATTTCCCATGATTCCTTCATATTT
GCATATACGATACAAGGCTGTTAGAGAGATAATTAGAATTAATTTGACTGTAAACACAAAGATATTAGT
ACAAAATACGTGACGTAGAAAGTAATAATTTCTTGGGTAGTTTGCAGTTTTAAAATTATGTTTTAAAAT
GGACTATCATATGCTTACCGTAACTTGAAAGTATTTCGATTTCTTGGCTTTATATATCTTGTGGAAAGG
ACGAAACACCGATGTTCCAATCAGTACGCAGTTTTAGTACTCTGGAAACAGAATCTACTAAAACAAGGC
AAAATGCCGTGTTTATCTCGTCAACTTGTTGGCGAGATTTTTTT

pU6-NmeCas9 sgRNA (U6 promoter-protospacer-NmeCas9 sgRNA scaffold-polyT)

CACATTTCCCCGAAAAGTGCCACCTGACGTCGCTAGCTGTACAAAAAAGCAGGCTTTAAAGGAACCAAT
TCAGTCGACTGGATCCGGTACCAAGGTCGGGCAGGAAGAGGGCCTATTTCCCATGATTCCTTCATATTT

GCATATACGATACAAGGCTGTTAGAGAGATAATTAGAATTAATTTGACTGTAAACACAAAGATATTAGT
ACAAAATACGTGACGTAGAAAGTAATAATTTCTTGGGTAGTTTGCAGTTTTAAAATTATGTTTTAAAAT
GGACTATCATATGCTTACCGTAACTTGAAAGTATTTCGATTTCTTGGCTTTATATATCTTGTGGAAAGG
ACGAAACACCGTTTAATGGTATAGAAGATCCAGTTGTAGCTCCCTTTCTCATTTCGGAAACGAAATGAG
AACCGTTGCTACAATAAGGCCGTCTGAAAAGATGTGCCGCAACGCTCTGCCCCTTAAAGCTTCTGCTTT
AAGGGGCATCGTTTATTTTTTT

pU6-St1Cas9 sgRNA (U6 promoter-protospacer-St1Cas9 sgRNA scaffold-polyT)
CACATTTCCCCGAAAAGTGCCACCTGACGTCGCTAGCTGTACAAAAAAGCAGGCTTTAAAGGAACCAAT
TCAGTCGACTGGATCCGGTACCAAGGTCGGGCAGGAAGAGGGCCTATTTCCCATGATTCCTTCATATTT
GCATATACGATACAAGGCTGTTAGAGAGATAATTAGAATTAATTTGACTGTAAACACAAAGATATTAGT
ACAAAATACGTGACGTAGAAAGTAATAATTTCTTGGGTAGTTTGCAGTTTTAAAATTATGTTTTAAAAT
GGACTATCATATGCTTACCGTAACTTGAAAGTATTTCGATTTCTTGGCTTTATATATCTTGTGGAAAGG
ACGAAACACCGTGAATCTGGTCAGAATATGATAAGGTTTTTGTACTCTCAAGATTTAAGTAACTGTACA
ACGAAACTTACACAGTTACTTAAATCTTGCAGAAGCTACAAAGATAAGGCTTCATGCCGAAATCAACAC
CCTGTCATTTTATGGCAGGGTGTTTTTTT

References

1 Wang H, Vilela M, Winkler A, Tarnawski M, Schlichting I, Yumerefendi H, Kuhlman B, Liu R, Danuser G, Hahn KM. LOVTRAP: an optogenetic system for photoinduced protein dissociation. *Nat Methods*. 2016;13:755–758.

2 Diensthuber RP, Engelhard C, Lemke N, Gleichmann T, Ohlendorf R, Bittl R, Möglich A. Biophysical, mutational, and functional investigation of the chromophore-binding pocket of light-oxygen-voltage photoreceptors. *ACS Synth Biol*. 2014;3:811–819.

3 Zayner JP, Sosnick TR. Factors that control the chemistry of the LOV domain photocycle. *PLoS One*. 2014;9:e87074.

4 Kay CW, Schleicher E, Kuppig A, Hofner H, Rüdiger W, Schleicher M, Fischer M, Bacher A, Weber S, Richter G. Blue light perception in plants. Detection and characterization of a light-induced neutral flavin radical in a C450A mutant of phototropin. *J Biol Chem*. 2003;278:10973–10982.

5 Harper SM, Christie JM, Gardner KH. Disruption of the LOV-Jalpha helix interaction activates phototropin kinase activity. *Biochemistry*. 2004;43:16184–16192.

6 Strickland D, Yao X, Gawlak G, Rosen MK, Gardner KH, Sosnick TR. Rationally improving LOV domain-based photoswitches. *Nat Methods*. 2010;7:623–626.

7 Strickland D, Lin Y, Wagner E, Hope CM, Zayner J, Antoniou C, Sosnick TR, Weiss EL, Glotzer M. TULIPs: tunable, light-controlled interacting protein tags for cell biology. *Nat Methods*. 2012;9:379–384.
